# SIVagm Infection in Wild African Green Monkeys from South Africa: Epidemiology, Natural History, and Evolutionary Considerations

**DOI:** 10.1371/journal.ppat.1003011

**Published:** 2013-01-17

**Authors:** Dongzhu Ma, Anna Jasinska, Jan Kristoff, J. Paul Grobler, Trudy Turner, Yoon Jung, Christopher Schmitt, Kevin Raehtz, Felix Feyertag, Natalie Martinez Sosa, Viskam Wijewardana, Donald S. Burke, David L. Robertson, Russell Tracy, Ivona Pandrea, Nelson Freimer, Cristian Apetrei, The International Vervet Research Consortium

**Affiliations:** 1 Center for Vaccine Research, University of Pittsburgh, Pittsburgh, Pennsylvania, United States of America; 2 Center for Neurobehavioral Genetics, Semel Institute for Neuroscience and Human Behavior, University of California Los Angeles, Los Angeles, California, United States of America; 3 Department of Genetics, Faculty of Natural and Agricultural Sciences, University of the Free State, Bloemfontein, South Africa; 4 Department of Anthropology, University of Wisconsin-Milwaukee, Milwaukee, Wisconsin, United States of America; 5 Computational and Evolutionary Biology, Faculty of Life Sciences, University of Manchester, Manchester, United Kingdom; 6 Departments of Pathology and Biochemistry, University of Vermont, Burlington, Vermont, United States of America; 7 Department of Pathology, University of Pittsburgh, Pittsburgh, Pennsylvania, United States of America; 8 Department of Microbiology and Molecular Genetics, University of Pittsburgh, Pittsburgh, Pennsylvania, United States of America; Harvard University, United States of America

## Abstract

Pathogenesis studies of SIV infection have not been performed to date in wild monkeys due to difficulty in collecting and storing samples on site and the lack of analytical reagents covering the extensive SIV diversity. We performed a large scale study of molecular epidemiology and natural history of SIVagm infection in 225 free-ranging AGMs from multiple locations in South Africa. SIV prevalence (established by sequencing *pol*, *env*, and *gag*) varied dramatically between infant/juvenile (7%) and adult animals (68%) (p<0.0001), and between adult females (78%) and males (57%). Phylogenetic analyses revealed an extensive genetic diversity, including frequent recombination events. Some AGMs harbored epidemiologically linked viruses. Viruses infecting AGMs in the Free State, which are separated from those on the coastal side by the Drakensberg Mountains, formed a separate cluster in the phylogenetic trees; this observation supports a long standing presence of SIV in AGMs, at least from the time of their speciation to their Plio-Pleistocene migration. Specific primers/probes were synthesized based on the *pol* sequence data and viral loads (VLs) were quantified. VLs were of 10^4^–10^6^ RNA copies/ml, in the range of those observed in experimentally-infected monkeys, validating the experimental approaches in natural hosts. VLs were significantly higher (10^7^–10^8^ RNA copies/ml) in 10 AGMs diagnosed as acutely infected based on SIV seronegativity (Fiebig II), which suggests a very active transmission of SIVagm in the wild. Neither cytokine levels (as biomarkers of immune activation) nor sCD14 levels (a biomarker of microbial translocation) were different between SIV-infected and SIV-uninfected monkeys. This complex algorithm combining sequencing and phylogeny, VL quantification, serology, and testing of surrogate markers of microbial translocation and immune activation permits a systematic investigation of the epidemiology, viral diversity and natural history of SIV infection in wild African natural hosts.

## Author Summary

We simultaneously assessed, for the first time in a natural host, the epidemiology, diversity and natural history of SIVagmVer infection in wild vervet populations from South Africa. We report that African green monkeys (AGMs) have likely been infected with SIVagm for a long period, ranging from the time of their speciation to Plio-Pleistocene migrations, refuting previous molecular clock calculations suggesting SIVagm to be of recent occurrence. As a result of virus-host coadaptation, SIVagmVer infection is characterized by a lack of disease progression in spite of robust viral replication. We show that very active SIVagm transmission in adult AGMs contrasts with a very limited transmission to their offspring, in spite of massive exposure to SIVagm both *in utero* and through breastfeeding. The observation that some AGMs remain uninfected in spite of life-long exposure to SIVagm identifies wild vervets as an acceptable animal model for the exposed uninfected individuals, which can be used to identify correlates of resistance to HIV/SIV infection.

## Introduction

Over 40 African nonhuman primate (NHP) species are naturally infected with simian immunodeficiency viruses (SIVs) [1]–[3]. Among these, African green monkeys (AGMs) (*Chlorocebus* genus) are the most numerous, most widely geographically spread and most commonly infected with SIV in the wild [1].

According to Groves [4], [5], AGMs are divided into species that are phenotypically and geographically distinct: vervets (*C. pygerythrus*) are the most widely spread in East and Southern Africa, ranging from the eastern Rift Valley in Ethiopia, Somalia and extreme southern Sudan to the Cape region in South Africa; grivets (*C. aethiops*) inhabit the area east of the White Nile in Ethiopia, Somalia from Khartoum to Mongalla, Eritrea and Ethiopia south to the Omo; tantalus monkeys (*C. tantalus*) are the prevalent species in north Central Africa from Ghana to Sudan and Kenya; green monkeys (*C. sabaeus*) reside in West Africa from Senegal to the Volta River, and have been introduced to Cape Verde Island as well as to the Caribbean [4]. A fifth AGM species, the malbrouck (*C. cynosuros*), is located in Central and South-Central Africa ranging from the Albertine Rift in the Democratic Republic of Congo to the Atlantic coast and Northern Namibia and Zambia. Finally, a sixth species, the Bale Mountain vervet (*C. djamdjamensis*) has a very limited distribution in the bamboo forests of highland Ethiopia. This taxonomic classification, however, is not universally accepted [6].

With the exception of the malbrouck and the Bale mountain vervet, each of the AGM species have been shown to be infected in the wild with species-specific SIVagm (defined as SIVagmVer, SIVagmGri, SIVagmTan and SIVagmSab) [7]–[11], with the reported prevalence rates ranging from30 to 50% [8], [12]–[19]. Phylogenetic analyses of SIVagm strains have shown that the spectrum of genetic diversity of SIVagm surpasses that of other primate lentiviruses [7], [9], [19]–[21], presumed to be due to codivergence/host-dependent evolution (i.e., the evolution of SIVagm from grivet, vervet, tantalus and green monkey species occurred in concert with host speciation) [8], [10], [11]. Together, these findings indicate that AGMs have coexisted with SIVagm for a long period of time, possibly even before their radiation and divergence from their common ancestor, approximately 1.5–3 million years ago [22].

Pathogenesis studies support an ancient coevolutionary relationship by showing that SIVagm are highly adapted to their hosts and that SIVagm-infected AGMs generally do not progress to AIDS [23], [24]. Key features of SIVagm infection in AGMs, as established by experimental studies performed on captive AGMs, include: (i) active viral replication, with set-point viral loads (VLs) similar to or higher than those found in HIV-infected patients [25]–[29]; (ii) significant depletion of CD4^+^ T cells during acute infection [25], [26], [30], [31], followed by rapid restoration to near preinfection levels in the peripheral blood [17], [25], [26], [30], [31] and delayed and incomplete restoration at mucosal sites [30]; (iii) maintenance of the balance between Th17 and T regulatory cells, due to preservation of the Th17 cell subset [32]; (iv) vigorous but transient inflammatory responses to the virus during acute infection, which are resolved with the transition from acute-to-chronic infection [30], [33], [34]; (v) productive infection of short-lived cells [31]; (vi) partial control of virus replication by the adaptive immune responses to SIV [35]–[41];(vii) no significant increase in CD4^+^ T cell apoptosis during either acute or chronic infection [30], [42], [43]. These normal levels of CD4^+^ T cell apoptosis together with preservation of Th17 cells probably allow AGMs to avoid enteropathy, breaches in the mucosal barrier and subsequent microbial translocation (MT) [30], as well as chronic immune activation and disease progression, while allowing CD4^+^ T cell recovery in the presence of high VLs [30], [44]. Furthermore, similar to other natural hosts of SIVs [45], AGMs have a number of adaptations that spare CD4^+^ T cells from virus-mediated killing *in vivo*. These features include a lower fraction of CD4^+^ T cells expressing the CCR5 coreceptor [46], [47] and down-regulation of the CD4 molecule by T helper cells as they enter the memory cell pool [48]–[50]. Altogether, these adaptations support the concept that the benign course of SIV infection in natural hosts is the result of coevolution over millennia [1], [51].

The ancestry of SIV infections and the codivergence of SIV types with their host species, however, could be accounted for by a model of preferential host-switching [52]. In the case of AGMs it has recently suggested that the phylogenetic profiles of SIVagm can be explained by a pattern of west-to-east transmission of the virus across existing AGM geographic ranges and that the viral divergence may have occurred more recently than previously suggested [53]. This study included only a small number of SIV strains covering a limited geographical distribution, which may have biased a full appreciation of the phylogenetic relationships and ancestry of SIVagm from different AGM species and likely altered the timing calculations for SIVagm emergence. A recent study in which calibration of molecular clocks was based on biogeographical features, reported that SIVs are actually older than estimated based on molecular clock calculations only [2].

The major focus of studies carried out thus far in wild African NHP species has been on identification and characterization of species-specific SIVs [54], [55]. A common denominator of these studies has been the difficulty of obtaining samples from wild animals and the lack of research infrastructure for pathogenesis studies in the field. Several approaches have been used to circumvent these limitations: (i) estimation of the SIV prevalence in captive monkeys, which significantly underestimates the prevalence in the wild, as most monkeys are captured at a young age and maternal-to-infant transmission rates are low in African NHP species [56]; (ii) use of bush meat samples. These are easy to obtain and thus this strategy has proven instrumental in identifying numerous SIVs and generating meaningful prevalence data [2], [57]–[62]. However, bush meat samples tend to be from adult populations and do not permit viral isolation or assessment of critical parameters of infection, such as VLs, immunophenotyping or assessment of the levels of immune activation; and (iii) use of noninvasive sampling (i.e., collection of fecal or urine samples), which has also been instrumental in assessing the prevalence of SIV infection in natural hosts, most notably in identifying and characterizing the animal reservoir of pandemic HIV strains [63]–[67]. However, similar to the use of bush meat samples, noninvasive samples cannot be used to assess key parameters of SIV infection. Thus, although SIVs have been identified and characterized for over two decades, studies of the natural history of SIV infection in the wild have only been performed at a cursory level [1], [54], [68].

Here, we performed for the first time in an African NHP host, a comprehensive large-scale characterization of the epidemiology and natural history of SIV infection in vervet monkeys living freely in different parks, nature reserves and farms from three provinces of the Republic of South Africa, to validate the virological and immunological features of experimental studies in natural hosts.

## Results

### Prevalence of retroviral infections in vervets from South Africa

**Figure 1:**
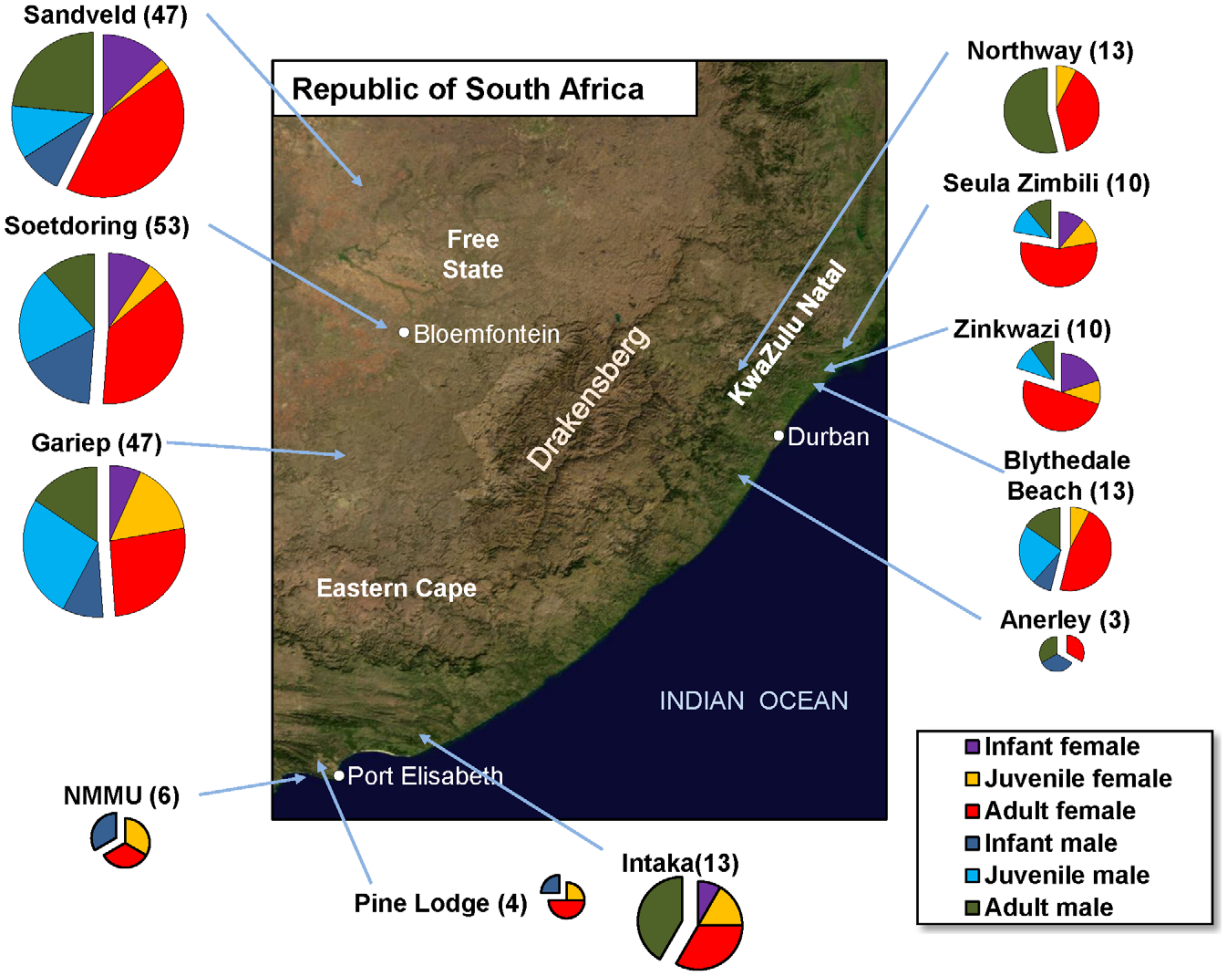
Satellite map of the Republic of South Africa indicating the geographical origin of the samples included in this study. Vervet samples from areas from three provinces (Free State, KwaZulu Natal and Eastern Cape) were included, as indicated. Sex and age-group coverage is illustrated.

Blood samples were collected from two hundred and twenty-five wild-trapped vervets over a 4.5 month period. Details on monkey capture and sample collection are presented as Supplementary information. The samples included represent 11 populations from 9 parks, game reserves and farms located in 3 provinces of South Africa–the Free State, KwaZulu-Natal, and Eastern Cape–covering majority significant proportion of vervet habitat in this region (Figure 1). Both sexes were equally represented. Samples from infants (<1 year old) and juvenile AGMs were included (Figure 1).

Diagnosis of SIVagmVer infection was performed by PCR, which allows for the evaluation of SIVagm diversity in addition to prevalence estimates. Also, in infants, PCR permits the diagnosis of infected offspring (as opposed to serological assays which are not specific, i.e., may also detect passively transmitted maternal antibodies). RNA was extracted from all plasma samples and subjected to RT-PCR analysis using consensus primers designed to amplify a 600-bp fragment in *pol* integrase and a 900-bp fragment in the gp120 gene encompassing the V3–V5 regions and the 5′ end of the gp41 gene. In addition, *gag* PCR (846 bp fragment in the p24 gene) was performed on selected samples from each location. This analysis identified 103 AGMs that were virion RNA (vRNA) positive using one, two or three primer sets (Table 1), giving an overall prevalence of SIVagmVer infection of 59% (73/123) in females and 29% (30/102) in males, in the range of those found in previous reports in AGMs [8], [15], [17]. Prevalence levels were relatively similar between different locations (Table S1) and higher than in a cohort of semifree animals (Table S2). SIVagm prevalence was very uneven in different age groups: 7% (3/44) in infants (males: 4%, 1/26; females: 11%, 2/18), 16% (9/58) in juveniles (males: 15%, 5/34; females: 21%, 4/21) and 71% (91/128) in adults (males: 57%, 24/42; females: 78%, 67/86). Thus, we confirmed on a very large number of samples previous results reporting that a dramatic increase in the SIVagm prevalence and incidence occurs with the passage to sexual maturity in AGMs [16]. Interestingly, and different from previous reports, we identified cases of SIVagmVer infection in very young AGMs, which may be suggestive of vertical transmission of the virus in the wild.

**Table 1:** Age- and sex-related prevalence of SIVagmVer in wild vervets from South Africa.

		Females			Males	
	Total	SIV+	Prevalence (%)	Total	SIV+	Prevalence (%)
Infants	18	2	11	26	1	4
Juveniles	19	4	21	34	5	15
Adults	86	67	78	42	24	57

### Genetic diversity of SIVagm in vervets from South Africa

**Figure 2:**
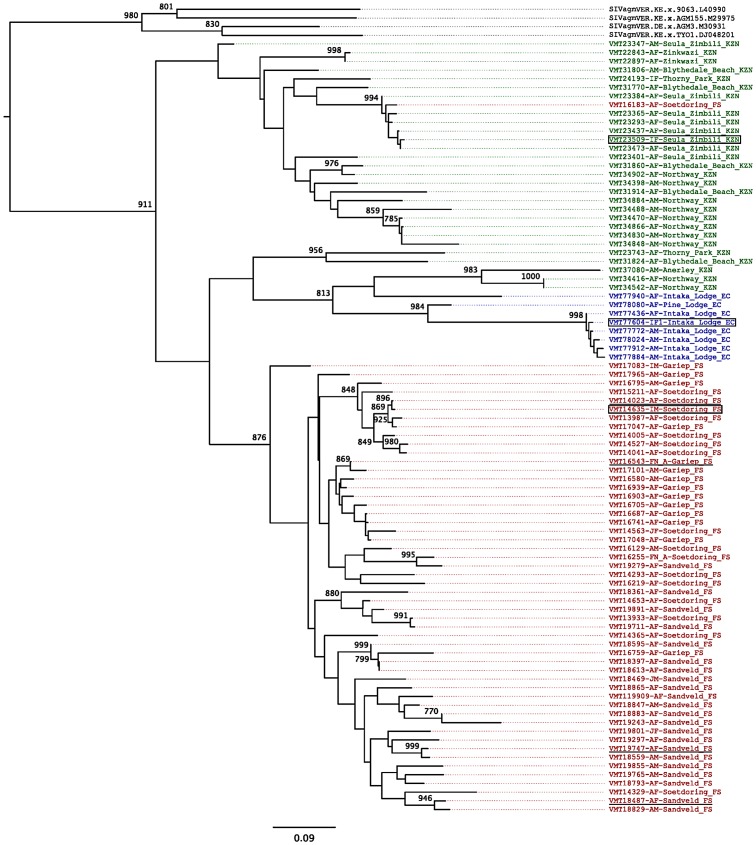
SIVagmVer diversity in wild AGMs from different parks and game reserves in the Republic of South Africa. Maximum likelihood tree for *pol* gene. Sequences are colored depending on the region in which the sequences were sampled, with red indicating sequences from Free State, green indicating sequences from KwaZulu-Natal, and blue indicating sequences from Eastern Cape territories. Reference SIVagm strains from vervet monkeys in Kenya are shown in black. The tree is based on a 592 bp *pol* sequence alignment (after gap-containing sites were removed). Maximum likelihood estimates were performed using 1000 replicate bootstrap analysis. Internal nodes indicate level of support values for internal branching. Bar represents number of amino acid replacements (0.09) per site. The strain nomenclature includes the identification number, monkey status (M-male, F-female; I-infant; J-juvenile and A-adult),and the site and state of origin (FS-Free State; KZN-KwaZulu Natal; EC-Eastern Cape). Putative heterosexual transmission cases documented to be of recent occurrence based on VL and serology testing are underlined. Putative maternal-to-infant transmission cases are boxed.

To determine the relationships of the newly identified SIVagm strains to each other and to previously characterized SIVagm strains, we constructed phylogenetic trees from amplified *pol* and *env* nucleotide sequences using a maximum likelihood approach (Figure 2 and Figure S1). While newly identified SIVagmVer strains naturally infecting vervets from South Africa exhibited a high genetic diversity, with average genetic distances in the *pol* gene of 16.2±4.8%, phylogenetic analyses also identified SIVagm strains that differed in less than 2% of their *pol* and *env* nucleotide sequences, indicating epidemiologically linked infections (Figure 2 and Figure S1). In general, strains originating from vervets from the same area clustered together (Figure 2) with a few exceptions that are probably due to male migration between groups when they reach sexual maturity.

As expected, phylogenetic analyses showed that SIVagmVer strains from South Africa clustered with the SIVagmVer reference strains (Figure 2 and Figure S1) and were only distantly related to the SIVagm strains from other AGM species (Figure 2). Furthermore, the newly characterized SIVagmVer strains from South Africa formed a subcluster within the SIVagmVer group, in agreement with the fact that all the reference strains available thus far have a distinct geographical origin, being collected from vervets in Kenya, Ethiopia and Tanzania [9], [19], [20]. Average genetic distances between the reference SIVagmVer strains (collected from Ethiopia, Kenya and Tanzania) and the newly characterized SIVagmVer strains were 25.4±2.1%, significantly higher than the overall genetic distances between South African strains (p<0.0001) (Table S3).

Similar to previous reports [66], comparison between the *pol* and *env* tree topologies showed numerous differences (Figure 2 and Figure S1), indicating extensive recombination within the SIVagm radiation (Figure S2). Mosaic branches occurred both near the tips of the trees and deeper in the trees, indicating that extensive recombination events have occurred throughout the evolutionary history of SIVagmVer [66]. This high frequency of recombinations between divergent SIVs indicates that SIVagm coinfection and/or superinfection occur frequently in wild vervets.

Remarkably, a geographically-associated clustering pattern can be defined for the South African SIVagmVer strains, with all but one of the SIVagmVer sequences from AGMs inhabiting different areas in the Free State forming a monophyletic cluster in the phylogenetic trees (Figure 2 and Figure S1).

Bootstrap analysis strongly supports a single origin of the Free State cluster, however parental nodes were associated with lower bootstrap support in the *pol* tree, suggesting ambiguity as to where the root of the Free State cluster should be placed. Lower bootstrap support may be indicative of recombination; a SplitsTree analysis shows that some KwaZulu-Natal sequences are likely to be recombinants of East Coast sequences, indicating a mixing between these two populations (Figure S2).

Interestingly, the SplitsTree analysis also identified one Free State sequence (VMT17083-IM-Gariep_FS) as potentially stemming from a distant recombination event with outgroup SIVagmVer sequences, this may suggest that this sequence is in fact a recombinant with an unknown parent (Figure S2).

**Figure 3:**
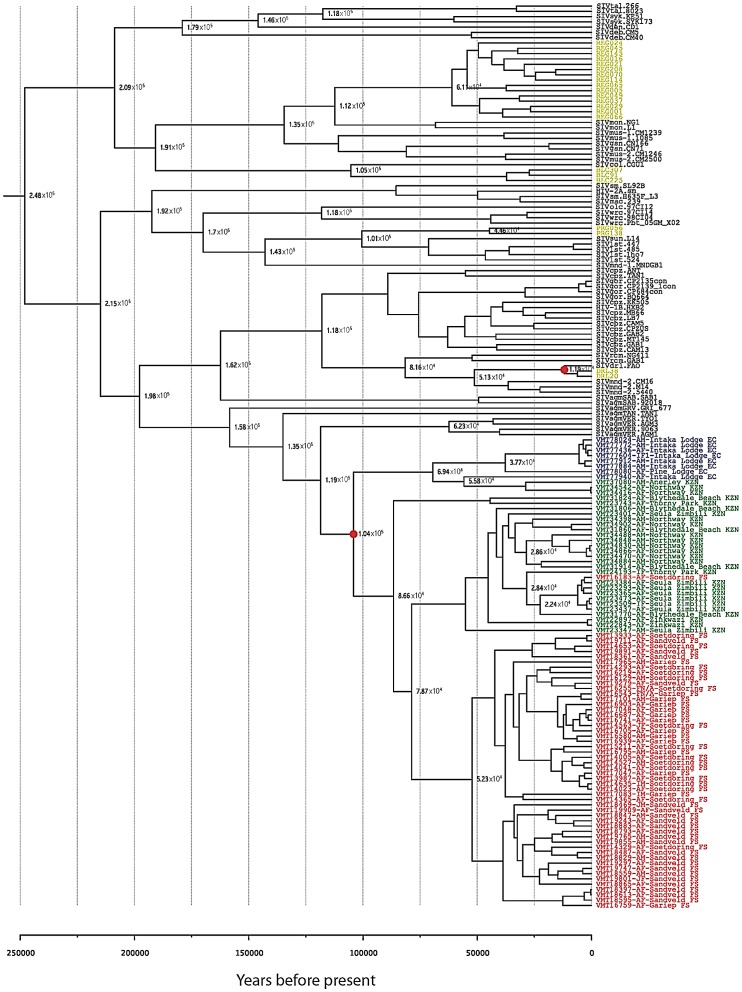
Molecular clock trees for *pol* gene based on the newly derived SIVagmVer sequences from vervets in South Africa. Relaxed molecular clock tree show the ultrametric divergence pattern between lineages. Calibration was based on internal nodes indicated with a red circle, by setting priors that span the presumed divergence MRCA date ranges, 100,000–3,000,000 years for SIVagm sequences surrounding the Drakensberg Mountains in South Africa, and 10,000±1,000 for SIVdrl sequences sampled from Bioko Island [2]. Internal nodes show divergence date estimates. Sequences are colored based on the territory from which they were sampled, with South African sequences colored red, green and blue, for Free State, KwaZulu Natal and Eastern Cape, respectively, and Bioko sequences colored in yellow.

The most likely explanation for these phylogenetic relationships is that the Drakensberg Mountains acted as a barrier to separate the vervet populations. We estimate that this separation could have occurred in a time frame ranging from 3 million years ago (during the AGM spread throughout sub-Saharan Africa) to 100,000 years ago (during the mass migrations that occurred in the Plio-Pleistocene glacial periods). We tested this hypothesis by calibrating a relaxed molecular clock to specify the time to the most recent ancestor (TMRCA) of the SIVagm samples surrounding the Drakensberg Mountains range to a time-frame spanning 100,000 to 3,000,000 years since the present for the *env* tree, and in the *pol* tree we additionally included SIV strains from several species and further calibrated the tree based on isolates from Bioko Island [2]. According to this analysis, divergence of the Free State lineage is likely to be closer to the 100,000 year mark, with mean most recent common ancestor (MRCA) for the South African SIVagm strains estimated at 329 thousands of years (kYa) (with 95% highest posterior density of 100–1,077 kYa) and 104 kYa (100–112 kYa), in the *env* tree (Table S4) and *pol* tree (Figure 3 and Table S4), respectively. This analysis places the root of the SIV tree older than previous studies [2], with the *env* tree giving a time estimate of SIV endemicity in simian species for 790 kYa (range: 151–2,595 kYa), and the *pol* tree indicating 248 kYa (range: 189–317 kYa) (Table S4).

### Analyses of the *env* gene

**Figure 4:**
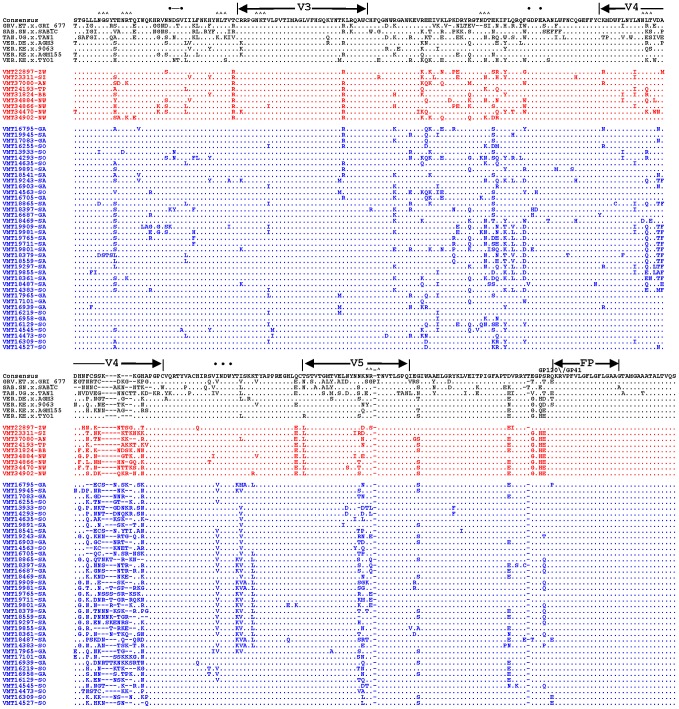
Alignment of partial envelope protein sequences from multiple SIVagmVer isolates. PCR-derived *env* nucleotide sequences were translated, aligned with previously reported SIVAGM *env* sequences [133] and compared with a consensus sequence generated by BioEdit. Dots denote sequence identity with the consensus sequence, while dashes represent gaps introduced to optimize the alignment. Triangles (∧) denote N-linked glycosylation sites. Bullets (•) mark CD4 binding sites. V3, V4 and V5 designate hypervariable SIVagm Env domains as previously described [10]. The envelope precursor cleavage site is indicated by an arrow above the consensus sequence. Strain nomenclature includes the identification number, monkey status (M-male, F-female; I-infant; J-juvenile and A-adult), and the site and the state of origin (FS-Free State; KZN-KwaZulu Natal; EC-Eastern Cape).

SIVagmVer envelope sequences (909 bp) encompassing the V3–V5 regions of the surface (SU) envelope glycoprotein and the 5′ end of the transmembrane (TM) domain were amplified by nested PCR from 48 plasma samples. Alignment of their deduced amino acid sequences with those of published reference SIVagm strains from all four species of AGMs identified hypervariable (V3–V5) as well as conserved envelope domains (Figure 4), as reported previously [10].

Regions corresponding to HIV-1 envelope domains of known function, including the envelope glycoprotein precursor cleavage site, the CD4 binding domain and the viral fusion peptide (N-terminus of gp41) were all highly conserved (Figure 4). Also conserved were the majority of cysteine residues (11 of 12) and, as reported previously [10], [18], there was very little sequence variability in the region that corresponds to the hypervariable V3 loop of HIV-1. Pairwise amino acid sequence comparisons were performed to examine the extent of SIVagmVer genetic diversity in the PCR amplified envelope fragment. As for the *pol* sequence analysis, *env* analysis revealed that SIVagmVer sequences collected from monkeys in the Free State form a distinct cluster from the monkeys living on coastal areas from both KwaZulu Natal and Eastern Cape provinces (Figure S1). This separate cluster was supported by both specific sequence signatures in the Free State-originating strains and by smaller genetic distances between these strains (Figure 4 and Figure S1).

Thus, sequence analysis identified: (i) a high diversity of SIVagm in South Africa, with the viruses collected from AGMs in the Free State forming an independent cluster, clearly separated from the strains collected from AGMs on the Indian Ocean coast; (ii) high frequency of recombination, suggestive of extensive transmission of the virus; and (iii) occurrence of highly related strains, suggestive of identifiable transmission clusters.

### Assessment of the natural history of SIVagmVer infection in wild AGMs

It is generally assumed that SIV infection is nonpathogenic in natural hosts [23], [51], [56]. However, this paradigm was recently challenged by studies demonstrating that SIVcpz has a substantial negative impact on the health, reproduction and lifespan of chimpanzees in the wild [68]–[70]. We therefore took advantage of this unique sample set to assess for the first time the natural history of SIV infection in a natural host monkey species in the wild.

### Clinical data

During sample collection, all included AGMs had a thorough clinical assessment. None of the vervets presented with any of the clinical signs associated with SIV infection, including fever, weight loss, lymphadenopathy or opportunistic infections. Although the cross-sectional nature of this study precludes assessment of changes in monkey weight and thus a direct assessment of weight loss, we performed comparative analyses of the body mass index (BMI) between SIV-positive and SIV-negative monkeys and showed that SIV status does not have an impact on normal weight of wild vervets (Figure S3).

### Genotypic assessment of the coreceptor usage of SIVagmVer strains from South Africa

Coreceptor usage by the different SIV strains may have a significant impact on the natural history of SIV infection in the wild. Previous studies reported that SIVagmSab strains have the ability to use the CXCR4 coreceptor for viral entry, in addition to CCR5 [26]. Since the amounts of plasma available from the AGMs in South Africa precluded *in vitro* phenotypic testing of coreceptor usage by these new strains, we estimated coreceptor use *in silico* by analyzing the V3 region. V3 sequence impacts SIVmac and SIVagm tropism [71]–[73].The algorithms for genotypic assessment of coreceptor usage based on the HIV-1 V3 sequences cannot be implemented for SIV [74], [75]. We therefore assessed the overall net charges of the V3 loop for these sequences and found that all the viruses exhibited the same V3 loop net charge (+7), similar to that of other SIV strains that are documented to use CCR5 [1]. No significant differences in the potential N-glycosylation sites located in the V3–V5 region could be documented for the newly derived SIVagmVer strains from South Africa (Figure 4). We therefore inferred that SIVagmVer strains identified here likely use CCR5 as the major coreceptor for entry, similar to the majority of SIV isolates [26], [40], [76].

### Staging of SIVagmVer infection in the wild: VL and serological testing

The levels of chronic viral replication are highly predictive of the outcome of HIV-1 infection [77], [78] and as we previously reported, may also predict the outcome of SIV infection in natural hosts, as the handful of African NHPs reported to progress to AIDS exhibited higher chronic VLs than nonprogressors [79]. To date, VLs in natural hosts of SIVs such as AGMs, sooty mangabeys and mandrills have only been assessed in either experimentally intravenously-infected monkeys [25], [80]–[84] or captive naturally infected monkeys [17], [28], [29], [79], [85], [86]. Only one study reported VLs in wild African monkeys on a very limited number of samples [85].

**Figure 5:**
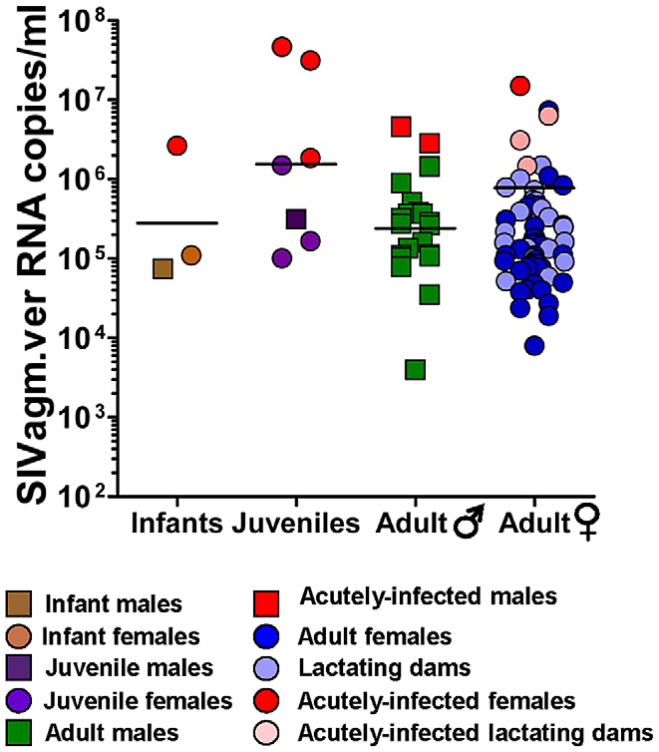
Cross-sectional analysis of SIVagm RNA levels in plasma of naturally infected AGM using a real-time RT-PCR assay. No significant difference could be observed between infant, juvenile and adult AGM or between female and male monkeys. Samples with high VLs assessed as acutely infected are shown. VLs of lactating dams are depicted in lighter colors. Detection limit: 100 copies/ml.

We used the generated SIVagmVer *pol* sequences to design specific primers and probes for viral quantification. The integrase region amplified by the *pol* primers used in our study is relatively well conserved; thus, the primers and probe had an excellent coverage of SIVagmVer diversity. We then quantified the VLs in 87 available samples from the 103 SIVagmVer-infected vervets. Results are shown in Figure 5. Plasma SIVagmVer VLs ranged from 10^4^ to 10^7^ copies/ml and were higher in the juvenile than in adult AGMs, albeit this difference was not statistically significant.

At least 23 of the 49 SIVagm-infected dams for which the VLs were available (47%) were documented as lactating at the time of sampling (Figure 5). The VLs of lactating dams are detailed in Figure 5 and demonstrate a large exposure of infant AGMs to SIV through breastfeeding.

While VLs of the majority of SIVagm-infected animals fell within a relatively close range, consistent with the range previously reported in experimentally infected African NHPs that are natural hosts of SIV [27], VLs were higher than expected in approximately 10% of vervets (Figure 5). Since such high VLs were observed in the majority of juvenile AGMs, we reasoned that they may be recent infections [27].

**Figure 6:**
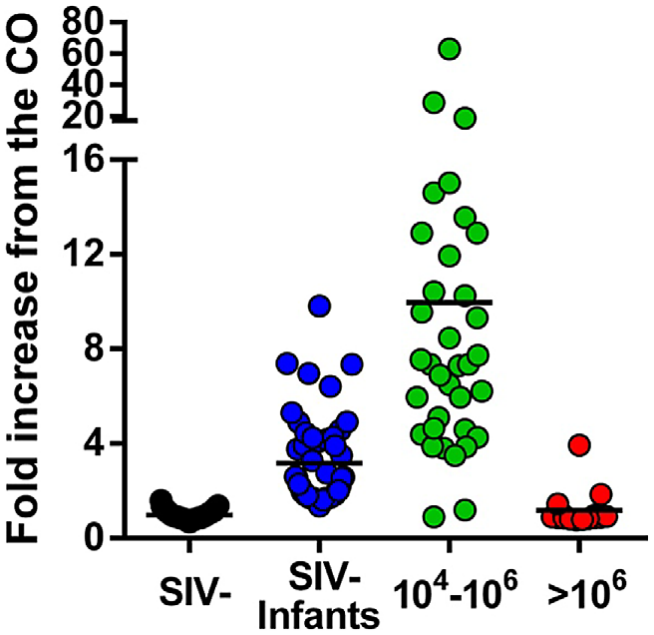
anti-SIVagmVer IgG antibody seroprevalence in vervets from South Africa. Testing was done by peptide ELISA (mapping the immunodominant region of gp41 of SIVagmVer). Samples were grouped based on PCR and VL quantification results as originating from uninfected vervets, SIV-infected vervets with VLs>10^6^ SIVagmVer RNA copies/ml, SIV-infected vervets with VLs ranging from 10^4^ to 10^6^ SIVagmVer RNA copies/ml and samples from infant vervets. Samples in the latter group are negative by PCR but show different levels of anti-SIVagm IgG, therefore being suggestive of transplacental crossing of maternal antibodies. Values on the Y-axis represent fold increases of the optical density of the samples (measured at 492 nm) over the cut-off value (established at an optical density of 0.2).

High levels of viral replication observed around the peak of VLs during acute HIV/SIV infection generally occur prior to seroconversion [87] and correspond to the Fiebeg II stage of HIV-1 infection [87]. Hence, to stage the SIVagm infection in AGMs with high VLs, we further designed a gp41 peptide ELISA and assessed the levels of anti-SIVagm antibodies in the available samples. As shown in Figure 6, the majority of SIVagm-infected AGMs harbored detectable levels of anti-gp41 antibodies. Conversely, most of the AGMs presenting with high VLs were seronegative. Altogether, these results strongly suggest that AGMs with high VLs were in the acute stage of infection [87]. Note, however, that definitive proof in support of this conclusion would rely on serial sampling and demonstration of seroconversion and partial control of viral replication [87].

Interestingly, serological testing showed that the majority (72%) of the plasma samples collected from infant AGMs were seropositive for SIVagm (Figure 6), while SIVagm could be amplified from only 3 of the infant samples (Figure 2 and Table 1), suggesting passive transfer of maternal antibodies and documenting massive *in utero* exposure of AGM offspring to SIVagm.

In conclusion, monitoring the viral replication and the anti-gp41 antibodies in SIVagmVer-infected AGMs allowed us to demonstrate that: (i) naturally occurring chronic SIV infection in a natural host is characterized by high VLs, which are in the same range as in experimentally-infected animals [27]; (ii) SIVagm transmission is very active in the wild; and (iii) the relatively low prevalence of SIVagm infection in offspring strongly contrasts with their massive *in utero* and breastfeeding exposure.

### Assessment of the integrity of mucosal barrier

**Figure 7:**
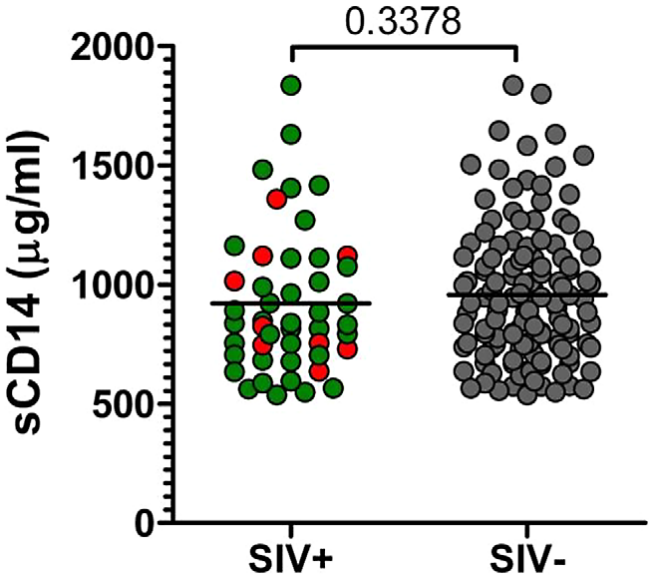
Plasma sCD14 levels in uninfected and SIVagmVer-infected vervets from South Africa. *P* value was calculated by the Mann-Whitney *U*-test. Animals diagnosed as acutely infected are denoted by light red circles.

Both SIV-infected AGMs and SMs were reported to maintain the integrity of mucosal barrier and thus to control MT [30], [84], which has been reported to be the main factor behind increased immune activation that drives disease progression in pathogenic HIV/SIV infections [88], [89]. To assess MT in wild vervets, we tested the levels of sCD14 as a surrogate biomarker. As shown in Figure 7, the levels of sCD14 were similar between SIV-infected and SIV-uninfected AGMs and were in the range of those we previously documented in captive uninfected and experimentally-infected AGMs. Even the samples collected from acutely infected monkeys did not show a significant increase in the levels of sCD14, in agreement with our previous results from captive experimentally infected AGMs [30], [90], and suggesting that wild SIVagm-infected AGMs maintain the integrity of the mucosal barrier.

### Cytokine and chemokine testing

**Figure 8:**
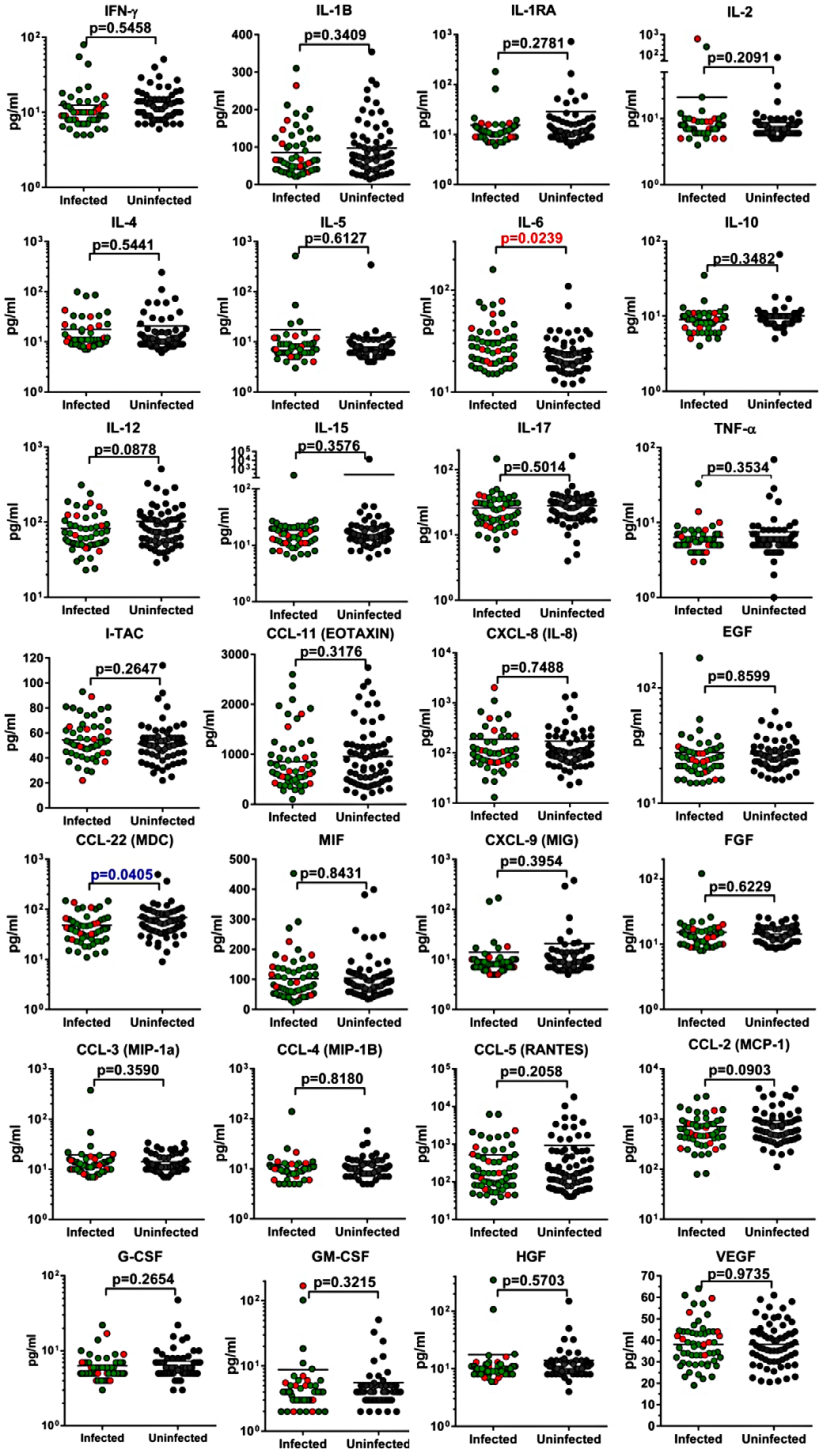
Cytokine and chemokine levels in uninfected and SIVagmVer-infected vervets from South Africa. *P* value was calculated by the Mann-Whitney *U*-test. Animals diagnosed as acutely infected are denoted by light red circles.

Finally, to assess the immune activation status between SIVagm-infected and uninfected AGMs in the wild, we measured and compared the levels of a large array of cytokines and chemokines in the available samples collected from the two groups in a Luminex assay. As shown in Figure 8, there was no significant increase in the cytokine/chemokine levels between SIV-infected and uninfected AGMs, with the exception of IL-6. In general, these cytokine/chemokine levels were in the same range of those previously reported by us and others [34] in captive uninfected and experimentally-infected AGMs during the chronic stage of SIVagm infection. This difference was mainly due to elevated levels of IL-6 in four of nine acutely-infected monkeys tested. When these samples were removed from the analysis, there were no significant differences in the levels of IL-6 between infected and uninfected AGMs. The increases in IL-6 probably reflect the transient increase in the levels of immune activation previously reported to occur during acute SIV infection in natural hosts [30], [33], [34], [91].

Thus, our analyses demonstrate the maintenance of the mucosal barrier and the lack of increased immune activation in acutely and chronically-infected AGMs in the wild. Immune activation being the major determinant of disease progression in pathogenic HIV/SIV infections, our results further support the clinical assessment of a benign outcome of SIV infection in wild AGMs.

## Discussion

AGMs are the largest reservoir of SIV in the wild [23] and one of the main animal models for the study SIV pathogenesis in natural hosts [23], [92]. However, so far no study has systematically investigated the prevalence and incidence of SIVagmVer in vervets and whether the natural history of SIVagm infection in wild free living animals differs from that of captive populations. To address these two key questions, we performed a large-scale analysis of SIVagmVer in wild vervets from the Republic of South Africa. Using only 0.5 ml of plasma and a new complex algorithm combining sequencing and phylogeny with virus quantification, serology, and assessment of surrogate markers of microbial translocation and immune activation, we concomitantly investigated, for the first time in a wild natural host African NHP monkey species, SIV diversity and evolution simultaneously with epidemiology and natural history. The strategy outlined here enables a systematic investigation of the natural history of SIV infection in wild natural hosts.

### Epidemiologic considerations

Vervets are abundant throughout East Africa along the Indian Ocean coast. However, most studies carried out thus far focused only on vervets from Northeast Africa (Kenya, Ethiopia and Tanzania) [14], and reported an SIVagmVer prevalence rate of 50% [17]. Conversely, our large-scale survey of SIVagm prevalence and diversity in vervets includes samples collected from all age groups and from multiple locations in South Africa. We confirm that the overall prevalence of SIVagm infection in the wild is high (46%), supporting the notion that SIVagm is uniformly distributed and widespread in AGMs [15], [16], [93]. These prevalence levels are similar to those previously reported for sooty mangabeys [66], [94], [95] and mandrills [96], which are among the most commonly infected NHP species known to date. For the first time, we document that one of the reasons for such extensive spread of SIV in wild vervets is the high steady-state SIVagm replication which probably facilitates virus transmission between AGMs through sexual contact [97].

SIVagm prevalence is higher in AGM females than in males. This difference is not surprising given that SIVagm transmission has lower efficacy through the foreskin compared to vaginal exposure [98] and that most males are expected to have significantly lower exposure, as only a few attain the dominance status necessary for sexual access to breeding females [99]. Note, however, that the robust levels of prevalence in males may be explained by the observation that despite the sexually restrictive nature of male dominance hierarchies in vervets, many subordinate males in multimale, multifemale groups can gain sexual access to females through a number of strategies [100]–[102], and thus become infected [103]. The relatively long breeding season, which lasts up to three months, may also reduce the ability of the alpha male to effectively monopolize fertile females, and increase the chances of transmission through sexual contact in subordinate males [104]. Furthermore, SIVagm transmission between males probably also occurs through aggressive, blood-exposing contacts for dominance, similar to other species [105], as many of the males sampled had open wounds or scars from such contact. Additionally, many females show marked aggression against dispersing males attempting to migrate into their social groups, often resulting in serious injury, providing another route of infection between males and infected females [106].

The very uneven distribution of SIVagm infection among different age groups strongly supports the sexual route as the main transmission route for SIVagm, together with a significantly less effective maternal-to-infant transmission. The low rates of mother-to-infant transmission are paradoxical, as our study documented large SIVagm exposure of the offspring both *in utero* and through breastfeeding. The rates of maternal-to-infant transmission in wild AGMs are significantly lower than the 35 to 40% mother-to-infant transmission rates reported in HIV infection (http://www.unaids.org) or 40–70% transmission rates in rhesus macaques [107] and are likely the result of virus-host coadaptation [97]. It is also possible that, similar to previous reports in captive sooty mangabeys and AGMs [108], [109], SIV infection of infant AGMs is associated with significantly lower levels of viremia than infection of adult monkeys, which may therefore go undetected by our assay.

Also conceivable is that, in addition to sexual and maternal-to-infant routes, a nonnegligible proportion of SIVagmVer transmissions occur through biting or fighting, as injuries are frequent (in our study sample, 10% of vervets showed signs of recent injuries that could result in exposure, such as deep lacerations). Injuries preponderantly occur during the mating season, due to both male transfer and to contests for dominance between males, and are usually cleaned by licking, which may involve many different individuals and may explain identification of near-identical viruses in vervets which are not direct mating partners. Studies have shown that monkeys are highly susceptible to SIV infection through oral exposure [110], [111], and the high levels of systemic viral replication documented in wild vervets may facilitate oral transmission during wound cleaning.

At least 10% of the cases of SIVagmVer infection in our group were recent infections (defined by high levels of viral replication close to the peak VL and negative serologies). Recent infections tend to occur in a narrow time frame (10–12 days postinoculation in experimentally infected macaques and African NHPs [27] and 3 weeks postinfection in HIV-1-infected patients [87]); therefore our results point to relatively high rates of SIVagm transmission in the wild. Note, however, that most transmissions are probably condensed in the time frame of the mating season, and thus the overall incidence of SIV infection in AGMs is not as high as suggested by our cross-sectional analysis.

### Virus diversity and evolutionary considerations

Our study identified a significant degree of divergence between strains collected from relatively homogenous groups of vervets, similar to previous reports for other species of African NHPs hosts, such as mandrills and sooty mangabeys [61], [66], [95], [96]. Also, we have identified closely related strains, suggestive of identifiable chains of transmission. In some instances, such highly related strains were observed in mother-offspring pairs, and probably occurred as a result of direct mother-to-infant transmission of the virus. In other instances, clusters of closely related viruses included a strain collected from a monkey documented as being recently infected. No clear association could be established for several of the closely related strains, and, as previously mentioned, these infections might have occurred during other social interactions, such as grooming or wound licking.

SIVagmVer strains from different locations were generally separated on the phylogenetic trees. Intermixing of strains was also observed, suggestive of SIVagm spread between communities by dispersing males. Indeed, although in vervet populations males range with their social group, they also migrate between groups upon or shortly after reaching sexual maturity (sometimes multiple times during the males lifetime) [106], [112].

Interestingly, the SIVagmVer strains collected from Free State locations (west of the Drakensberg Mountains) clustered differently within *pol* and *env* trees (Figure 2 and S1) as well as in the *gag* tree (data not shown) from those collected from monkeys situated along the Indian Ocean coast (east of the Drakensberg Mountains). Given that the geographical spread between coastal monkeys and those from Free State is similar, the most likely explanation for these phylogenetic relationships is that the Drakensberg Mountains acted as a barrier (through low temperatures in the elevated areas) to separate the vervet populations on the two sides. The Drakensberg Mountains are very old, with an estimated age of 280 million years, which implies that a separation of the vervet populations could have occurred in a time frame ranging from 3 million years ago (during the AGM spread throughout sub-Saharan Africa) to 100,000 years go (during the mass migrations that occurred in the Plio-Pleistocene glacial periods). The Drakensberg Mountains may also have acted as an effective barrier to separate different populations of at least two other species, the common impala (*Aepyceros melampus melampus*) [113] and the chacma baboon (*Papio ursinus*) [114]. We therefore performed relaxed molecular clock analyses to specify TMRCA of the SIVagm samples surrounding the Drakensberg Mountains range to a time-frame spanning 100,000 to 3,000,000 years since the present for the *env* tree, and in the *pol* tree we additionally included SIV strains of several species and further calibrated the tree based on isolates from Bioko Island [2]. Molecular clock analysis using Bayesian techniques is highly dependent on priors that are specified [115]; however our analysis is in agreement with another study in which the use of biogeography showed that SIVs are very old [2], and refuted timing calculations based exclusively on molecular clocks without regards to biogeographical calibration points [53].

Taken together, these observations strongly suggest that SIVagm infection of vervets is more ancient than the most recent previous estimate [53] and suggests that the concept of host-dependent evolution (supported by the observation that the four SIVagm types cluster together in the SIV trees) cannot be discarded using current molecular clock calibrations, but may possibly be discernible through the integration of further biogeographical divergence estimates.

### Natural history considerations

It is generally assumed that SIV infection is nonpathogenic in natural hosts [1], [23], [44], [51], [54]. Data supporting this assumption are derived from the study of captive monkeys and a very limited number of species (AGMs, sooty mangabeys and mandrills) [1], [23], [44], [51]. Sporadic cases of progression to AIDS were reported in each of these species upon infection with their species-specific viruses [116]–[118] or with cross-species transmitted SIVs [119]. Large scale studies in wild animals are needed to draw definitive conclusions about pathogenicity of SIVs in their natural hosts in the wild, as demonstrated by recent observation that SIVcpz infection is pathogenic in naturally-infected chimpanzees [68], [69], [120]. The fact that such a major effect went undetected for decades supports the need for systematic assessment of the natural history of SIV infection in African NHPs in their native habitat, and not in captive environments (where health status is controlled, nutrition is monitored and exposure to adventitious agents that may impact clinical status is limited), to provide significant information regarding the clinical outcome of SIV infection in natural hosts.

The main limitation of our assessment of natural history of SIVagmVer infection in vervets is that only plasma samples were available, and thus we could not perform immunophenotypic characterization or *in vitro* replication studies. We circumvented this limitation by employing a combination of biomarkers that could be monitored in plasma.

We report that SIVagmVer infection most likely has a benign outcome in vervets. BMI were not different between SIV-infected and SIV-uninfected vervets. Detected levels of viral replication were high, but in the range of those reported during experimental studies [27]. In the documented cases of acute SIVagmVer infection, the VL levels were also in the range of those observed in experimentally infected monkeys during acute infection [27].

We measured levels of sCD14 to assess the levels of MT, which is one of the proposed mechanisms of excessive immune activation that drives HIV/SIV infection to progress to AIDS [88], [89]. sCD14 is a surrogate marker for direct measurement of endotoxin or Gram negative bacteria, and numerous studies have shown a strong association of increasing sCD14 levels and increasing LPS levels in plasma in both pathogenic HIV and SIV infections (presumably due to loss of barrier function in the gut in turn due to HIV-mediated destruction of GALT) [88], [89], and lack of increase in both markers in the setting of nonpathogenic SIV infections in natural hosts [30], [84], [90], [121].

It is critical to assess the integrity of the mucosal barrier in a natural host in the wild, as we and others have previously reported that lack of MT during chronic infection in AGMs and sooty mangabeys may be one mechanism of controlling chronic immune activation and disease progression [30], [84]. These studies have been done using captive NHPs. Wild monkeys are exposed to many pathogens in a significantly more hostile environment than are captive animals, which may impact the ability of SIV-infected monkeys to maintain the barrier. However, we found no significant difference in the levels of sCD14 between infected and uninfected vervets in the wild.

In addition to being a surrogate marker of MT, sCD14 is a marker of macrophage activation, and thus our results also suggest that there are no significant differences in the levels of macrophage activation between SIV-infected and uninfected AGMs in the wild.

Lack of chronic immune activation was also supported by the similar levels of cytokines and chemokines tested in SIVagmVer-infected and uninfected wild vervets. The only exception was represented by an increased level of IL-6 in SIVagmVer-infected animals, which was driven by high levels of this cytokine in acutely infected monkeys. We and others reported that a transient increase in immune activation occurs during acute SIV infection in natural hosts [33], [35], [36], [91], [122], which is rapidly resolved with the transition to chronic infection [30]. Therefore, the similar levels of cytokine in plasma between SIV-infected and uninfected animals are somewhat surprising in the context of a significant number of acute infections in our study sample. This apparent discrepancy with the results of experimental studies may be due to the fact that most of the experimental studies involved intravenous administration of large amounts of virus that may trigger inflammation and immune activation. Alternatively, as we have previously shown, the acute increases in levels of plasma cytokines and chemokines are very transient in natural hosts, being thus likely that our cross-sectional samples missed such increases.

Altogether, sCD14 and cytokine/chemokine testing data support the conclusion that SIVagmVer infection of wild vervets is not associated with significant increases in levels of immune activation. Immune activation being the main driver of HIV disease progression, our results corroborate the clinical assessment and support a nonprogressive outcome of SIVagmVer infection in the wild.

### Final considerations: Wild vervet populations from South Africa as an animal model for exposed-seronegative/exposed-uninfected (ESN/EU) patients

Both clinical experience and a growing medical literature indicate that some persons who have been exposed to HIV infection remain uninfected [123]. Although in some instances this may simply represent good fortune, cohorts of uninfected persons have been reported who are considered at high risk for infection. Clarifying the determinants of protection against HIV infection is a high priority that will require careful selection of high-risk uninfected cohorts, which should undergo targeted studies of plausible mediators and broad screening for unexpected determinants of protection. An animal model for ESN/EU may permit such an assessment while circumventing the major ethical boundaries to the study of ESN/EU humans. Such an animal model is not currently available. The SIVmac/RM model is not relevant for ESN/EU studies because this model was derived for pathogenesis and vaccine studies (thus employing strains selected for pathogenicity and not for their transmission characteristics) [124]. On the other hand, *de novo* development of such a model in RMs would be extremely expensive and face major macaque availability restrictions. In this context, the SIVagmVer-infected wild vervet cohorts that we identified in South Africa may serve as a useful model for ESN/EU studies. In these populations, there is an enormous exposure to SIVagm. However, while up to 80% of sexually-active adult females are infected with SIVagmVer, the remaining 20% are uninfected in spite of the presumably massive potential for exposure to SIV throughout their lives. Thus, field studies to assess the prevalence and incidence of SIV infection in the wild and prospectively confirm resistance to infection in a subset of ESN/EU monkeys, combined with genetic, immunologic and virologic assessment of the monkeys in the cohorts, may permit identification of correlates of ESN/EU, an instrumental leap for designing new therapeutic strategies aimed at preventing HIV infection.

In conclusion, we report that SIVagmVer is highly prevalent in wild vervet populations from South Africa, that the virus has long been present in this population and that coevolution between SIVagmVer and its natural host, the vervet monkey, resulted in an infection which has a benign outcome in the wild. More importantly, our study has identified a potential model for the study of ESN/EU cases, which has a strong potential to identify correlates of protection against HIV/SIV infection.

## Materials and Methods

### Ethics statement

All the animals sampled in this study were used according to regulations set forth by the Animal Welfare Act. The animal sampling protocols were approved by the University of Wisconsin-Milwaukee Institutional Animal Care and Use Committee (IACUC). Studies at the UCLA and University of Pittsburgh were carried out through an agreement with the UWM. At the University of the Free State UFS, ethical clearance was provided by the Interfaculty Animal Ethics Committee (project no. 13/2010).

### Samples

The study was carried out in feral and semifree vervets (*n* = 225) living in different areas in South Africa, aged between 6 months and 10 years (Figure 1). Animals were individually trapped using established methods [125]. Details on animal capture are provided as Text S1. Blood was collected through venous puncture. Details are provided as Text S1. Each monkey included in the study had a microchip implanted for further identification and to prevent duplicate sampling. Detailed clinical assessment of the monkeys (including signs associated with immunodeficiency, i.e., lymphadenopathy, low weight, fever or rashes) was performed at the time of blood collection. Details are provided as Text S1. Upon collection, plasma was immediately stored at −80°C. A 500 µl aliquot of plasma from each monkey was available for this study.

### Plasma processing and testing strategy

Upon arrival, plasma samples were thawed for RNA extraction and separated into three aliquots: the first aliquot was used for testing the prevalence levels of SIV by PCR. Additional SIV ELISA testing was performed using this aliquot to establish the stage of acute SIV infection and infant exposure to SIVagm. The second aliquot was used for quantification of SIVagm VLs by real-time PCR. Primers and probes were designed based on the SIVagm integrase sequences. Correlation between VLs and serology enabled identification of acutely SIV-infected AGMs [87]. Finally, the third aliquot was used to assess the natural history of SIVagm infection in the wild by testing surrogate markers for microbial translocation and immune activation (sCD14 and cytokine/chemokine levels).

### Viral RNA extraction and cDNA synthesis

From each plasma specimen, viral RNA was extracted using the QIAamp Viral RNA Mini kit (QIAGEN, Germantown, MD). RNA was eluted and immediately subjected to cDNA synthesis. Reverse transcription of RNA to single-stranded cDNA was performed using SuperScript III reverse transcription according to the manufacturer's recommendations (Invitrogen, Carlsbad, CA). In brief, each cDNA reaction included 1×RT buffer, 0.5 mM of each deoxynucleoside triphosphate, 5 mM dithiothreitol, 2 U/ml RNaseOUT (RNase inhibitor), 10 U/ml of Super-Script III reverse transcriptase, and 0.25 µM antisense primers SIV-POL-OR (5′-ACB ACY GCN CCT TCH CCT TTC-3′), SIV-GAG-B (5′-CCT ACT CCC TGA CAG GCC GTC AGC ATT TCT TC-3′) and SIV-ENV-B (5′-AGA GCT GTG ACG CGG GCA TTG AGG TT-3′). The mixture was incubated at 50°C for 60 min, followed by an increase in temperature to 55°C for an additional 60 min. The reaction was then heat-inactivated at 70°C for 15 min and treated with 2 U of RNase H at 37°C for 20 min. The newly synthesized cDNA was used immediately or frozen at −80°C.

### Nested PCR and DNA gel extraction

SIVagm RNA was amplified byPCR using Platinium Taq Polymerase High Fidelity (Invitrogen, Carlsbad, CA). A 600 bp *pol* integrase fragment was amplified by nested PCR using outer primers POL-IS4 (5′-CCA GCN CAC AAA GGN ATA GGA GG-3′) and POL-OR and inner primers SIV-POL-IS4 and SIV-POL-Unipol2 (5′-CCC CTA TTC CTC CCC TTC TTT TAA AA-3′) [59], [126]. An 846 bp *gag* fragment was amplified by nested PCR using outer primers GAG-A (5′-AGG TTA CGG CCC GGC GGA AAG AAA A-3′) and GAG-B, and inner primers GAG-C (5′-AGT ACA TGT TAA AAC ATG TAG TAT GGG C-3′) and GAG-F (5′-CCT TAA GCT TTT GTA GAA TCT ATC TAC ATA-3′) [127]. Finally, a 900-bp e*nv* fragment encompassing the V3-V5 gp120 region and the 5′ end of gp41 envelope regions was amplified by nested PCR using outer primers ENV-A (5′-GAA GCT TGT GAT AAA ACA TAT TGG AT-3′) and ENV-B, and inner primers ENV-C (5′-GTG CAT TGT ACA GGG TTA ATG AAT ACA ACA G-3′) and ENV-D (5′-TTC TTC TGC TGC AGTA TCC CAG CAA G-3′) [128]. Nested PCR products were subjected to 1% agarose (Invitrogen, Carlsbad, CA) gel electrophoresis and extracted and purified by QIAquick Gel Extraction kit (QIAGEN, Germantown, MD).

### DNA sequencing

SIV gene amplicons were directly sequenced by the University of Pittsburgh Genomics and Proteomics Core laboratories (GPCL) using the nested PCR primers. Individual sequence for each amplicon was edited and inspected using BioEdit 7.1.3. Sequences were translated for analyses on the V3–V5 region of the SIVagmVer envelope with BioEdit.

### Phylogenetic analysis

The *gag*, *pol* and *env* nucleotide sequence alignments were aligned with reference sequences obtained from the Los Alamos National Laboratory HIV Sequence Database (http://hiv-web.lanl.gov). The alignment for the *pol* region included nucleotide sequences sampled from the island of Bioko [2]. Newly derived SIV sequences were aligned using MUSCLE sequence alignment software [129] and alignments were edited manually where necessary.

Separate phylogenetic trees for each of the genes were inferred by maximum-likelihood using PhyML 3.0 [130], with a GTR substitution model, gamma-distributed rate heterogeneity at sites and an SPR tree search. Nonparametric bootstrap analysis with 1,000 replicates was performed to assess the reliability of branching order. In addition, calibrated molecular clock trees were calculated using BEAST v1.7.3 [115] to infer likely MRCA dates between different lineages. For molecular clock analysis, we used an HKY substitution model with gamma-distributed rate heterogeneity among sites and an uncorrelated lognormal relaxed molecular clock. A Yule tree prior was used for the *pol* region, and a constant size coalescent for the *env* region. Evolutionary rate was calibrated by setting a uniform prior bounded by 10,000 and 3,000,000 years, as TMRCA of SIVagm isolates sampled from areas surrounding the Drakensberg Mountains. For the *pol* region, a normal distribution with a mean of 10,000 years and a standard deviation of 1,000 years was additionally used to calibrate TMRCA of Bioko and mainland SIVdrl sequences. Chains were run for 10^7^–10^8^ states and ESS values were high (>300). Analysis of the molecular clock trees was performed using Tracer v1.5 (http://tree.bio.ed.ac.uk/software/tracer/), and trees were inferred by maximum clade credibility with mean node heights using TreeAnnotator v1.7.3, and visualized using FigTree v1.3.1 (http://tree.bio.ed.ac.uk/software/figtree/). To test for signals of recombination in the dataset, we ran the Neighbor-Net algorithm implemented in SplitsTree [131] to infer phylogenetic networks for the *env* and *pol* regions.

### Viral load quantification

We used the generated *pol* alignment to design specific primers and probe for VL quantification in wild vervets: pol-F (5′-GTA GCC AGT GGG TTC ATA GAA GCA-3′), pol-R (5′-CAT TGC CTT TAC TTC CTG TGA AAT GA-3′) and pol-Probe (5′-/56-FAM/TAG AGA AAC/ZEN/AGG AAA AGA AAC AGC AA/3IABkF/-3′). Primers and probe were synthesized by IDTDNA (Coralville, IA) and were used in a one-step real-time PCR assay with TaqMan One Step PCR Master Mix (Applied Biosystems, Branchburg, NJ). Real-time PCR was performed in MicroAmp Optical 96-well plates (Applied Biosystems, Branchburg, NJ) by mixing the OneStep RT-PCR Enzyme Mix with 5 µl isolated RNA in a 50 µl final reaction volume. Real-time PCR conditions were as follows: 30 min at 50°C, 15 min at 95°C, followed by 45 cycles of 95°C for 15 s and 60°C for 1 min. Dilutions of all components were made using sterile RNase-free water. Data were collected and analyzed using the PE Applied Biosystems software provided. RNA copies/well were adjusted to copies per milliliter of original plasma. Samples were tested in duplicate and the number of RNA copies determined by comparison with a standard curve obtained using known amounts of SIV-pol RNA. The SIVagm RNA standard was produced by cloning one of the SIVagmVer*pol* gene fragments into Vector pBluescript SK+ and linearizing it by Xba I as a DNA template for RNA *in vitro* transcription. Standard RNA was produced by using MEGAscript kit (Applied Biosystems, Branchburgh, NJ), according to the manufacturer's instructions. Briefly, the transcription reaction system was assembled at room temperature and mixed thoroughly, followed by incubation at 37°C for 4 hours. One microliter of TURBO DNase was added to the reaction mixture followed by incubation at 37°C for 15 minutes to digest the DNA template. RNA was recovered by Lithium chloride precipitation. The amount of RNA was quantified at A260, aliquoted and immediately stored at −80°C.Detection limit of the SIVagmVer quantification assay was 100 copies/ml.

### Serology

Based on the *env* sequence data, a peptide mapping the immunodominant region in the Gp41 transmembrane glycoprotein was synthesized and used in a peptide enzyme linked immunosorbent assay (ELISA), as described [132]. The inferred peptide sequence was TALEKYLEDQARLNVWGCAWKQVC, and this sequence was very well conserved between different SIVagmVer isolates, including previously reported reference strains from Kenya and Ethiopia [9], [10], [93]. The Gp36 peptide was synthesized to a purity of at least 90% (Fisher Scientific, USA) and the assay was performed as previously reported [132]. The cut-off of the reaction was arbitrarily set at 0.20, as per previous reports [132].

### Microbial translocation (MT)

MT was assessed using the levels of *soluble CD14 (sCD14)* as a surrogate marker [89]. CD14 is a transmembranous protein, which also exists in a soluble form (sCD14; both as a shed membrane form and an alternatively spliced form), as a part of the complex that presents endotoxin (lipopolysaccharide or LPS) to TLR4 on monocytes. When monocytes are activated, ectodomain shedding results in increased sCD14 levels. sCD14 is therefore surrogate for direct measurement of endotoxin or Gram negative bacteria which translocate from the intestinal lumen to general circulation as a result of the immunologic injury inflicted at the mucosal level by the pathogenic HIV/SIV infection [88]–[90], [121]. sCD14 levels were measured using a quantitative sandwich enzyme immunoassay technique (Quantikine Human sCD14 Immunoassay, R&D Systems, Minneapolis, MN). The detection limit of this kit is 200 ng/mL and can range up to 5000 ng/mL, with an interassay coefficient of variability ranging between 7.19% and 10.9%.

### Cytokine and chemokine testing

Cytokine testing in plasma was done using a sandwich immunoassay-based protein array system, Cytokine Monkey Magnetic 28-Plex Panel (Invitrogen, Camarillo, CA), as instructed by the manufacturer, and results were read by the Bio-Plex array reader (Bio-Rad Laboratories, Hercules, CA), which uses Luminex fluorescent-bead-based technology (Luminex Corporation, Austin, TX).

### Statistical analyses

Statistical analyses were performed to analyze the results of viral load, sCD14 and cytokine testing using Mann-Whitney *U*-tests and Prism 4.0 software (Prism, Irvine, CA).

### Nucleotide sequence accession numbers

The nucleotide sequences of the *pol* and *env* sequences from SIVagmVer-infected vervets from South Africa were deposited in the GeneBank (accession numbers: JX462308-JX462444).

## Supporting Information

Figure S1 **Maximum likelihood trees for** ***env*** **gene of the newly derived SIVagmVer sequences from wild vervets in South Africa.** Maximum likelihood estimates were performed using 1000 replicate bootstrap analysis. Internal nodes indicate level of support values for internal branching. Sequences are colored depending on the region in which the sequences were sampled, with red indicating sequences from Free State, green indicating sequences from KwaZulu-Natal, and blue indicating sequences from Eastern Coast territories. In addition, sequences colored in gray are sampled from the Riverside Wildlife Rehabilitation and Environmental Education Centre (RWREC), Letsitele, Limpopo semifree colony that is housing released pets or animals with health problems, and their origin is unknown, and sequences colored in black indicate outgroup sequences. The strain nomenclature includes the identification number, monkey status (M-male, F-female; I-infant; J-juvenile and A-adult), and the site and the state of origin (FS-Free State; KZN-KwaZulu Natal; EC-Eastern Cape).
(TIFF)

Figure S2 **SplitsTree showing the pol gene of South African SIVagmVer sequences.** Nodes are colored by the region in which they were sampled, with red, blue and green denoting Free State, East Coast and KwaZulu-Natal, respectively. Potentially interesting recombinants have been circled, including VMT17083-IM-Gariep_FS and a cluster indicating possible mixing between East Coast and KwaZulu-Natal sequences.
(TIFF)

Figure S3 **Comparative body mass index (BMI) assessment based on age and location in SIV-infected and SIV uninfected wild vervet monkeys (** ***Chlorocebus pygerithrus*** **) from South Africa.**
(TIFF)

Table S1 **SIV prevalence in vervet monkeys from South Africa based on location of collected samples.**
(DOC)

Table S2 **Age- and sex-related prevalence of SIVagmVer in semifree vervets from Riverside Wildlife Rehabilitation and Education Center, Letsitele, Limpopo.**
(DOC)

Table S3 **Estimates of evolutionary divergence over sequence pairs between groups.** The number of base substitutions per site±standard error estimate(s) from averaging over all sequence pairs between groups are shown± Analyses were conducted using the Maximum Composite Likelihood model [1]. The rate variation among sites was modeled with a gamma distribution (shape parameter = 0.5). *env* analysis involved 81 nucleotide sequences. All positions containing gaps and missing data were eliminated. There were a total of 822 positions in the final dataset for *env*. *pol* analysis involved 98 nucleotide sequences. All positions containing gaps and missing data were eliminated. There were a total of 574 positions in the final *pol* dataset. Evolutionary analyses were conducted in MEGA5 [2].
(DOC)

Table S4 **Most recent common ancestor (MRCA) estimates using relaxed molecular clocks to incorporate biogeographical assumptions.** Calibration for the *env* gene is based on time to MRCA (TMRCA) of SIVagm sequences sampled around the Drakensberg Mountains, presumed to be 100,000–3,000,000 years of age. Calibration of the *pol* gene is based on TMRCA of SIVagm sequences as before, as well as SIVdrl sequences sampled from the Bioko Island of Equatorial Guinea, presumed to have been isolated for approximately 10,000 years. These estimates indicate that SIV is potentially considerably older than previous estimates, and refute timing estimates based exclusively on molecular clocks without calibration.
(DOC)

Text S1 **Details on animal origin, animal capture, sampling methodology and clinical assessment.**
(DOC)

## References

[1] VandeWoude S, Apetrei C (2006) Going wild: Lessons from T-lymphotropic naturally occurring lentiviruses. Clin Microbiol Rev 19: 728–762. 17041142 10.1128/CMR.00009-06 PMC1592692

[2] Worobey M, Telfer P, Souquière S, Hunter M, Coleman CA, et al (2010) Island biogeography reveals the deep history of SIV. Science 330: 1487. 20847261 10.1126/science.1193550

[3] Ahuka-Mundeke S, Ayouba A, Mbala-Kingebeni P, Liegeois F, Esteban A, et al (2011) Novel multiplexed HIV/simian immunodeficiency virus antibody detection assay. Emerg Infect Dis 17: 2277–2286. 22172157 10.3201/eid1712.110783 PMC3311211

[4] Groves C (2001) Primate taxonomy. Washington, DC: Smithsonian Institution Press.

[5] Groves CP (2005) Order Primates. In: Wilson DE, Reeder DM, editors. Mammal Species of the World. Baltimore, MD: The Johns Hopkins University Press. pp. 111–184.

[6] Grubb P, Butinsky TM, Oates JF, Bearder SK, Disotell TD, et al (2003) Assessment of the diversity of African primates. Int J Primatol 24: 1301–1357.

[7] Fomsgaard A, Allan J, Gravell M, London WT, Hirsch VM, et al (1990) Molecular characterization of simian lentiviruses from east African green monkeys. J Med Primatol 19: 295–303. 2231686

[8] Hirsch VM, McGann C, Dapolito G, Goldstein S, Ogen-Odoi A, et al (1993) Identification of a new subgroup of SIVagm in tantalus monkeys. Virology 197: 426–430. 8212578 10.1006/viro.1993.1606

[9] Fukasawa M, Miura T, Hasegawa A, Morikawa S, Tsujimoto H, et al (1988) Sequence of simian immunodeficiency virus from African green monkey, a new member of the HIV/SIV group. Nature 333: 457–461. 3374586 10.1038/333457a0

[10] Jin MJ, Hui H, Robertson DL, Muller MC, Barre-Sinoussi F, et al (1994) Mosaic genome structure of simian immunodeficiency virus from west African green monkeys. EMBO J 13: 2935–2947. 8026477 10.1002/j.1460-2075.1994.tb06588.x PMC395175

[11] Allan JS, Short M, Taylor ME, Su S, Hirsch VM, et al (1991) Species-specific diversity among simian immunodeficiency viruses from African green monkeys. J Virol 65: 2816–2828. 2033656 10.1128/jvi.65.6.2816-2828.1991 PMC240900

[12] Hendry RM, Wells MA, Phelan MA, Schneider AL, Epstein JS, et al (1986) Antibodies to simian immunodeficiency virus in African green monkeys in Africa in 1957–62. Lancet 2: 455. 2874433 10.1016/s0140-6736(86)92156-2

[13] Lowenstine LJ, Pedersen NC, Higgins J, Pallis KC, Uyeda A, et al (1986) Seroepidemiologic survey of captive Old-World primates for antibodies to human and simian retroviruses, and isolation of a lentivirus from sooty mangabeys (*Cercocebus atys*). Int J Cancer 38: 563–574. 2428760 10.1002/ijc.2910380417

[14] Ohta Y, Masuda T, Tsujimoto H, Ishikawa K, Kodama T, et al (1988) Isolation of simian immunodeficiency virus from African green monkeys and seroepidemiologic survey of the virus in various non-human primates. Int J Cancer 41: 115–122. 2447023 10.1002/ijc.2910410121

[15] Phillips-Conroy JE, Jolly CJ, Petros B, Allan JS, Desrosiers RC (1994) Sexual transmission of SIVagm in wild grivet monkeys. J Med Primatol 23: 1–7. 7932633 10.1111/j.1600-0684.1994.tb00088.x

[16] Jolly C, Phillips-Conroy JE, Turner TR, Broussard S, Allan JS (1996) SIVagm incidence over two decades in a natural population of Ethiopian grivet monkeys (*Cercopithecus aethiops aethiops*). J Med Primatol 25: 78–83. 8864978 10.1111/j.1600-0684.1996.tb00198.x

[17] Goldstein S, Brown CR, Ourmanov I, Pandrea I, Buckler-White A, et al (2006) Comparison of simian immunodeficiency virus SIVagmVer replication and CD4+ T-cell dynamics in vervet and sabaeus African green monkeys. J Virol 80: 4868–4877. 16641278 10.1128/JVI.80.10.4868-4877.2006 PMC1472054

[18] Muller MC, Saksena NK, Nerrienet E, Chappey C, Herve VM, et al (1993) Simian immunodeficiency viruses from central and western Africa: evidence for a new species-specific lentivirus in tantalus monkeys. J Virol 67: 1227–1235. 8437214 10.1128/jvi.67.3.1227-1235.1993 PMC237488

[19] Johnson PR, Fomsgaard A, Allan J, Gravell M, London WT, et al (1990) Simian immunodeficiency viruses from African green monkeys display unusual genetic diversity. J Virol 64: 1086–1092. 2304139 10.1128/jvi.64.3.1086-1092.1990 PMC249221

[20] Baier M, Garber C, Muller C, Cichutek K, Kurth R (1990) Complete nucleotide sequence of a simian immunodeficiency virus from African green monkeys: a novel type of intragroup divergence. Virology 176: 216–221. 2158689 10.1016/0042-6822(90)90246-n

[21] Li Y, Naidu Y, Fultz P, Daniel MD, Desrosiers RC (1989) Genetic diversity of simian immunodeficiency virus. J Med Primatol 18: 261–269. 2569537

[22] Xing J, Wang H, Zhang Y, Ray DA, Tosi AJ, et al (2007) A mobile element-based evolutionary history of guenons (tribe Cercopithecini). BMC Biol 5: 5. 17266768 10.1186/1741-7007-5-5 PMC1797000

[23] Pandrea I, Apetrei C (2010) Where the wild things are: Pathogenesis of SIV infection in African nonhuman primate hosts. Curr HIV/AIDS Reports 7: 28–36. 20425055 10.1007/s11904-009-0034-8 PMC2824118

[24] Pandrea I, Silvestri G, Apetrei C (2009) AIDS in African nonhuman primate hosts of SIVs: A new paradigm of SIV infection. Curr HIV Res 6: 57–72. 10.2174/157016209787048456 19149555

[25] Pandrea I, Apetrei C, Dufour J, Dillon N, Barbercheck J, et al (2006) Simian immunodeficiency virus (SIV) SIVagm.sab infection of Caribbean African green monkeys: New model of the study of SIV pathogenesis in natural hosts. J Virol 80: 4858–4867. 16641277 10.1128/JVI.80.10.4858-4867.2006 PMC1472068

[26] Pandrea I, Kornfeld C, Ploquin MJ-I, Apetrei C, Faye A, et al (2005) Impact of viral factors on very early *in vivo* replication profiles in SIVagm-infected African green monkeys. J Virol 79: 6249–6259. 15858009 10.1128/JVI.79.10.6249-6259.2005 PMC1091729

[27] Pandrea I, Silvestri G, Onanga R, Veazey RS, Marx PA, et al (2006) Simian immunodeficiency viruses replication dynamics in African non-human primate hosts: common patterns and species-specific differences. J Med Primatol 35: 194–201. 16872282 10.1111/j.1600-0684.2006.00168.x

[28] Broussard SR, Staprans SI, White R, Whitehead EM, Feinberg MB, et al (2001) Simian immunodeficiency virus replicates to high levels in naturally infected African green monkeys without inducing immunologic or neurologic disease. J Virol 75: 2262–2275. 11160730 10.1128/JVI.75.5.2262-2275.2001 PMC114810

[29] Goldstein S, Ourmanov I, Brown CR, Beer BE, Elkins WR, et al (2000) Wide range of viral load in healthy african green monkeys naturally infected with simian immunodeficiency virus. J Virol 74: 11744–11753. 11090174 10.1128/jvi.74.24.11744-11753.2000 PMC112457

[30] Pandrea I, Gautam R, Ribeiro R, Brenchley JM, Butler IF, et al (2007) Acute loss of intestinal CD4+ T cells is not predictive of SIV virulence. J Immunol 179: 3035–3046. 17709518 10.4049/jimmunol.179.5.3035 PMC2367134

[31] Pandrea I, Ribeiro RM, Gautam R, Gaufin T, Pattison M, et al (2008) Simian immunodeficiency virus SIVagm dynamics in African green monkeys. J Virol 82: 3713–3724. 18216122 10.1128/JVI.02402-07 PMC2268485

[32] Favre D, Lederer S, Kanwar B, Ma ZM, Proll S, et al (2009) Critical loss of the balance between Th17 and T regulatory cell populations in pathogenic SIV infection. PLoS Pathogens 5: e1000295. 19214220 10.1371/journal.ppat.1000295 PMC2635016

[33] Jacquelin B, Mayau V, Targat B, Liovat AS, Kunkel D, et al (2009) Nonpathogenic SIV infection of African green monkeys induces a strong but rapidly controlled type I IFN response. J Clin Invest 119: 3544–3555. 19959873 10.1172/JCI40093 PMC2786805

[34] Kornfeld C, Ploquin MJ, Pandrea I, Faye A, Onanga R, et al (2005) Antiinflammatory profiles during primary SIV infection in African green monkeys are associated with protection against AIDS. J Clin Invest 115: 1082–1091. 15761496 10.1172/JCI23006 PMC1062895

[35] Gaufin T, Pattison M, Gautam R, Stoulig C, Dufour J, et al (2009) Effect of B cell depletion on viral replication and clinical outcome of SIV infection in a natural host. J Virol 83: 10347–10357. 19656874 10.1128/JVI.00880-09 PMC2753117

[36] Gaufin T, Ribeiro RM, Gautam R, Dufour J, Mandell D, et al (2010) Experimental depletion of CD8+ cells in acutely SIVagm-infected African green monkeys results in increased viral replication. Retrovirology 7: 42. 20459829 10.1186/1742-4690-7-42 PMC2879233

[37] Zahn RC, Rett MD, Korioth-Schmitz B, Sun Y, Buzby AP, et al (2008) Simian Immunodeficiency Virus (SIV)-specific CD8+ T cell responses in chronically SIVagm-infected vervet African green monkeys. J Virol 82: 11577–11588. 18829748 10.1128/JVI.01779-08 PMC2583661

[38] Zahn RC, Rett MD, Li M, Tang H, Korioth-Schmitz B, et al (2010) Suppression of adaptive immune responses during primary SIV infection of sabaeus African green monkeys delays partial containment of viremia but does not induce disease. Blood 115: 3070–3078. 20147699 10.1182/blood-2009-10-245225 PMC2858477

[39] Schmitz JE, Zahn RC, Brown CR, Rett MD, Li M, et al (2009) Inhibition of adaptive immune responses leads to a fatal clinical outcome in SIV-infected pigtailed macaques but not vervet African green monkeys. PLoS Pathog 5: e1000691. 20011508 10.1371/journal.ppat.1000691 PMC2785481

[40] Hirsch VM (2004) What can natural infection of African monkeys with simian immunodeficiency virus tell us about the pathogenesis of AIDS? AIDS Rev 6: 40–53. 15168740

[41] Gicheru MM, Otsyula M, Spearman P, Graham BS, Miller CJ, et al (1999) Neutralizing antibody responses in Africa green monkeys naturally infected with simian immunodeficiency virus (SIVagm). J Med Primatol 28: 97–104. 10475110 10.1111/j.1600-0684.1999.tb00257.x

[42] Estaquier J, Idziorek T, de Bels F, Barre-Sinoussi F, Hurtrel B, et al (1994) Programmed cell death and AIDS: significance of T-cell apoptosis in pathogenic and nonpathogenic primate lentiviral infections. Proc Natl Acad Sci U S A 91: 9431–9435. 7937784 10.1073/pnas.91.20.9431 PMC44826

[43] Cumont MC, Diop O, Vaslin B, Elbim C, Viollet L, et al (2008) Early divergence in lymphoid tissue apoptosis between pathogenic and nonpathogenic simian immunodeficiency virus infections of nonhuman primates. J Virol 82: 1175–1184. 18032487 10.1128/JVI.00450-07 PMC2224460

[44] Pandrea I, Sodora DL, Silvestri G, Apetrei C (2008) Into the wild: simian immunodeficiency virus (SIV) infection in natural hosts. Trends Immunol 29: 419–428. 18676179 10.1016/j.it.2008.05.004 PMC2840226

[45] Paiardini M, Cervasi B, Reyes-Aviles E, Micci L, Ortiz AM, et al (2011) Reduced CCR5 up-regulation upon activation limits virus replication in central-memory CD4^+^ T cells of SIV-infected sooty mangabeys. Nature Medicine 17: 830–836. 10.1038/nm.2395 PMC3253129 21706028

[46] Pandrea I, Apetrei C, Gordon S, Barbercheck J, Dufour J, et al (2007) Paucity of CD4+CCR5+ T cells is a typical feature of natural SIV hosts. Blood 109: 1069–1076. 17003371 10.1182/blood-2006-05-024364 PMC1785133

[47] Pandrea I, Onanga R, Souquiere S, Mouinga-Ondéme A, Bourry O, et al (2008) Paucity of CD4+CCR5+ T-cells may prevent breastfeeding transmission of SIV in natural non-human primate hosts. J Virol 82: 5501–5509. 18385229 10.1128/JVI.02555-07 PMC2395173

[48] Beaumier CM, Harris LD, Goldstein S, Klatt NR, Whitted S, et al (2009) CD4 downregulation by memory CD4+ T cells *in vivo* renders African green monkeys resistant to progressive SIVagm infection. Nat Med 15: 879–885. 19525963 10.1038/nm.1970 PMC2723181

[49] Vinton C, Klatt NR, Harris LD, Briant JA, Sanders-Beer BE, et al (2011) CD4-like immunological function by CD4- T cells in multiple natural hosts of simian immunodeficiency virus. J Virol 85: 8702–8708. 21715501 10.1128/JVI.00332-11 PMC3165829

[50] Apetrei C, Gaufin T, Gautam R, Vinton C, Hirsch VM, et al (2010) Pattern of SIVagm infection in patas monkeys suggests that host adaptation to SIV infection may result in resistance to infection and virus extinction. J Infect Dis 202 Suppl 3: S371–376. 20887227 10.1086/655970 PMC2951294

[51] Silvestri G, Paiardini M, Pandrea I, Lederman MM, Sodora DL (2007) Understanding the benign nature of SIV infection in natural hosts. J Clin Invest 117: 3148–3154. 17975656 10.1172/JCI33034 PMC2045617

[52] Charleston MA, Robertson DL (2002) Preferential host switching by primate lentiviruses can account for phylogenetic similarity with the primate phylogeny. Syst Biol 51: 528–535. 12079649 10.1080/10635150290069940

[53] Wertheim JO, Worobey M (2007) A challenge to the ancient origin of SIVagm based on African green monkey mitochondrial genomes. PLoS Pathog 3: e95. 17616975 10.1371/journal.ppat.0030095 PMC1904472

[54] Hahn BH, Shaw GM, De Cock KM, Sharp PM (2000) AIDS as a zoonosis: scientific and public health implications. Science 287: 607–614. 10649986 10.1126/science.287.5453.607

[55] Peeters M, Courgnaud V, Abela B, Auzel P, Pourrut X, et al (2002) Risk to human health from a plethora of simian immunodeficiency viruses in primate bushmeat. Emerg Infect Dis 8: 451–457. 11996677 10.3201/eid0805.01-0522 PMC2732488

[56] Apetrei C, Robertson DL, Marx PA (2004) The history of SIVS and AIDS: epidemiology, phylogeny and biology of isolates from naturally SIV infected non-human primates (NHP) in Africa. Front Biosci 9: 225–254. 14766362 10.2741/1154

[57] Courgnaud V, Abela B, Pourrut X, Mpoudi-Ngole E, Loul S, et al (2003) Identification of a new simian immunodeficiency virus lineage with a vpu gene present among different cercopithecus monkeys (*C. mona*, C. cephus, and *C*. *nictitans*) from Cameroon. J Virol 77: 12523–12534. 14610175 10.1128/JVI.77.23.12523-12534.2003 PMC262559

[58] Courgnaud V, Formenty P, Akoua-Koffi C, Noe R, Boesch C, et al (2003) Partial molecular characterization of two simian immunodeficiency viruses (SIV) from African colobids: SIVwrc from Western red colobus (*Piliocolobus badius*) and SIVolc from olive colobus (*Procolobus verus*). J Virol 77: 744–748. 12477880 10.1128/JVI.77.1.744-748.2003 PMC140619

[59] Courgnaud V, Pourrut X, Bibollet-Ruche F, Mpoudi-Ngole E, Bourgeois A, et al (2001) Characterization of a novel simian immunodeficiency virus from guereza colobus monkeys (*Colobus guereza*) in Cameroon: a new lineage in the nonhuman primate lentivirus family. J Virol 75: 857–866. 11134299 10.1128/JVI.75.2.857-866.2001 PMC113982

[60] Courgnaud V, Salemi M, Pourrut X, Mpoudi-Ngole E, Abela B, et al (2002) Characterization of a novel simian immunodeficiency virus with a vpu gene from greater spot-nosed monkeys (*Cercopithecus nictitans*) provides new insights into simian/human immunodeficiency virus phylogeny. J Virol 76: 8298–8309. 12134035 10.1128/JVI.76.16.8298-8309.2002 PMC155126

[61] Apetrei C, Metzger MJ, Robinson D, Ling B, Telfer PT, et al (2005) Detection and partial characterization of new simian immunodeficiency virus (SIVsm) strains from bush meat samples from rural Sierra Leone. J Virol 79: 2631–2636. 15681464 10.1128/JVI.79.4.2631-2636.2005 PMC546599

[62] Liegeois F, Courgnaud V, Switzer WM, Murphy HW, Loul S, et al (2006) Molecular characterization of a novel simian immunodeficiency virus lineage (SIVtal) from northern talapoins (*Miopithecus ogouensis*). Virology 349: 55–65. 16469345 10.1016/j.virol.2006.01.011

[63] Keele BF, Van Heuverswyn F, Li Y, Bailes E, Takehisa J, et al (2006) Chimpanzee reservoirs of pandemic and nonpandemic HIV-1. Science 313: 523–526. 16728595 10.1126/science.1126531 PMC2442710

[64] Van Heuverswyn F, Li Y, Neel C, Bailes E, Keele BF, et al (2006) Human immunodeficiency viruses: SIV infection in wild gorillas. Nature 444: 164. 17093443 10.1038/444164a

[65] Santiago ML, Lukasik M, Kamenya S, Li Y, Bibollet-Ruche F, et al (2003) Foci of endemic simian immunodeficiency virus infection in wild-living eastern chimpanzees (*Pan troglodytes schweinfurthii*). J Virol 77: 7545–7562. 12805455 10.1128/JVI.77.13.7545-7562.2003 PMC164799

[66] Santiago ML, Range F, Keele BF, Li Y, Bailes E, et al (2005) Simian immunodeficiency virus infection in free-ranging sooty mangabeys (*Cercocebus atys atys*) from the Tai Forest, Cote d'Ivoire: implications for the origin of epidemic human immunodeficiency virus type 2. J Virol 79: 12515–12527. 16160179 10.1128/JVI.79.19.12515-12527.2005 PMC1211554

[67] Santiago ML, Rodenburg CM, Kamenya S, Bibollet-Ruche F, Gao F, et al (2002) SIVcpz in wild chimpanzees. Science 295: 465. 11799233 10.1126/science.295.5554.465

[68] Keele BF, Holland Jones J, Terio K, Estes JD, Rudicell RS, et al (2009) Increased mortality and AIDS-like immunopathology in wild chimpanzees infected with SIVcpz. Nature 460: 515–519. 19626114 10.1038/nature08200 PMC2872475

[69] Rudicell RS, Holland Jones J, Wroblewski EE, Learn GH, Li Y, et al (2010) Impact of simian immunodeficiency virus infection on chimpanzee population dynamics. PLoS Pathog 6: e1001116 doi: 10.1371/journal.ppat.1001116. 20886099 10.1371/journal.ppat.1001116 PMC2944804

[70] Terio KA, Kinsel MJ, Raphael J, Mlengeya T, Lipende I, et al (2011) Pathologic lesions in chimpanzees (*Pan trogylodytes schweinfurthii*) from Gombe National Park, Tanzania, 2004–2010. J Zoo Wildl Med 42: 597–607. 22204054 10.1638/2010-0237.1 PMC3693847

[71] Kirchhoff F, Morrison HG, Desrosiers RC (1995) Identification of V3 mutations that can compensate for inactivating mutations in C4 of simian immunodeficiency virus. Virology 213: 179–189. 7483261 10.1006/viro.1995.1558

[72] Konig RR, Flory E, Steidl S, Neumann J, Coulibaly C, et al (2002) Engineered CD4- and CXCR4-using simian immunodeficiency virus from African green monkeys is neutralization sensitive and replicates in nonstimulated lymphocytes. J Virol 76: 10627–10636. 12368305 10.1128/JVI.76.21.10627-10636.2002 PMC136611

[73] Steidl S, Stitz J, Schmitt I, Konig R, Flory E, et al (2002) Coreceptor Switch of [MLV(SIVagm)] pseudotype vectors by V3-loop exchange. Virology 300: 205–216. 12350351 10.1006/viro.2001.1565

[74] Recordon-Pinson P, Soulie C, Flandre P, Descamps D, Lazrek M, et al (2010) Evaluation of the genotypic prediction of HIV-1 coreceptor use versus a phenotypic assay and correlation with the virological response to maraviroc: the ANRS GenoTropism study. Antimicrob Agents Chemother 54: 3335–3340. 20530226 10.1128/AAC.00148-10 PMC2916345

[75] Lengauer T, Sander O, Sierra S, Thielen A, Kaiser R (2007) Bioinformatics prediction of HIV coreceptor usage. Nat Biotechnol 25: 1407–1410. 18066037 10.1038/nbt1371

[76] Gautam R, Carter AC, Katz N, Butler IF, Barnes M, et al (2007) *In vitro* characterization of primary SIVsmm isolates belonging to different lineages. *In vitro* growth on rhesus macaque cells is not predictive for *in vivo* replication in rhesus macaques. Virology 362: 257–270. 17303205 10.1016/j.virol.2006.12.037 PMC1936220

[77] Mellors JW, Munoz A, Giorgi JV, Margolick JB, Tassoni CJ, et al (1997) Plasma viral load and CD4+ lymphocytes as prognostic markers of HIV-1 infection. Ann Intern Med 126: 946–954. 9182471 10.7326/0003-4819-126-12-199706150-00003

[78] Mellors JW, Rinaldo CR Jr, Gupta P, White RM, Todd JA, et al (1996) Prognosis in HIV-1 infection predicted by the quantity of virus in plasma. Science 272: 1167–1170. 8638160 10.1126/science.272.5265.1167

[79] Apetrei C, Gautam R, Sumpter B, Carter AC, Gaufin T, et al (2007) Virus-subtype specific features of natural SIVsmm infection in sooty mangabeys. J Virol 81: 7913–7923. 17507488 10.1128/JVI.00281-07 PMC1951324

[80] Diop OM, Gueye A, Dias-Tavares M, Kornfeld C, Faye A, et al (2000) High levels of viral replication during primary simian immunodeficiency virus SIVagm infection are rapidly and strongly controlled in African green monkeys. J Virol 74: 7538–7547. 10906207 10.1128/jvi.74.16.7538-7547.2000 PMC112274

[81] Onanga R, Kornfeld C, Pandrea I, Estaquier J, Souquiere S, et al (2002) High levels of viral replication contrast with only transient changes in CD4+ and CD8+ cell numbers during the early phase of experimental infection with simian immunodeficiency virus SIVmnd-1 in *Mandrillus sphinx* . J Virol 76: 10256–10263. 12239301 10.1128/JVI.76.20.10256-10263.2002 PMC136548

[82] Onanga R, Souquiere S, Makuwa M, Mouinga-Ondeme A, Simon F, et al (2006) Primary simian immunodeficiency virus SIVmnd-2 infection in mandrills (*Mandrillus sphinx*). J Virol 80: 3303–3309. 10.1128/JVI.80.7.3301-3309.2006 PMC1440382 16537597

[83] Silvestri G, Fedanov A, Germon S, Kozyr N, Kaiser WJ, et al (2005) Divergent host responses during primary simian immunodeficiency virus SIVsm infection of natural sooty mangabey and nonnatural rhesus macaque hosts. J Virol 79: 4043–4054. 15767406 10.1128/JVI.79.7.4043-4054.2005 PMC1061583

[84] Gordon S, Klatt NR, Milush JM, Engram J, Dunham RM, et al (2007) Severe depletion of mucosal CD4+ T cells in AIDS-free SIV-infected sooty mangabeys. J Immunol 179: 3026–3034. 17709517 10.4049/jimmunol.179.5.3026 PMC2365740

[85] Pandrea I, Onanga R, Kornfeld C, Rouquet P, Bourry O, et al (2003) High levels of SIVmnd-1 replication in chronically infected *Mandrillus sphinx* . Virology 317: 119–127. 14675630 10.1016/j.virol.2003.08.015

[86] Silvestri G, Sodora DL, Koup RA, Paiardini M, O'Neil SP, et al (2003) Nonpathogenic SIV infection of sooty mangabeys is characterized by limited bystander immunopathology despite chronic high-level viremia. Immunity 18: 441–452. 12648460 10.1016/s1074-7613(03)00060-8

[87] Fiebig EW, Wright DJ, Rawal BD, Garrett PE, Schumacher RT, et al (2003) Dynamics of HIV viremia and antibody seroconversion in plasma donors: implications for diagnosis and staging of primary HIV infection. AIDS 17: 1871–1879. 12960819 10.1097/00002030-200309050-00005

[88] Brenchley JM, Price DA, Douek DC (2006) HIV disease: fallout from a mucosal catastrophe? Nat Immunol 7: 235–239. 16482171 10.1038/ni1316

[89] Brenchley JM, Price DA, Schacker TW, Asher TE, Silvestri G, et al (2006) Microbial translocation is a cause of systemic immune activation in chronic HIV infection. Nature Medicine 12: 1365–1371. 10.1038/nm1511 17115046

[90] Pandrea I, Gaufin T, Brenchley JM, Gautam R, Monjure C, et al (2008) Experimentally-induced immune activation in natural hosts of SIV induces significant increases in viral replication and CD4+ T cell depletion. J Immunol 181: 6687–6691. 18981083 10.4049/jimmunol.181.10.6687 PMC2695139

[91] Bosinger SE, Li Q, Gordon SN, Klatt NR, Duan L, et al (2009) Global genomic analysis reveals rapid control of a robust innate response in SIV-infected sooty mangabeys. J Clin Invest 119: 3556–3572. 19959874 10.1172/JCI40115 PMC2786806

[92] Muller MC, Barre-Sinoussi F (2003) SIVagm: genetic and biological features associated with replication. Front Biosci 8: D1170–1185. 12957815 10.2741/1130

[93] Johnson PR, Gravell M, Allan J, Goldstein S, Olmsted RA, et al (1989) Genetic diversity among simian immunodeficiency virus isolates from African green monkeys. J Med Primatol 18: 271–277. 2547962

[94] Apetrei C, Kaur A, Lerche NW, Metzger M, Pandrea I, et al (2005) Molecular epidemiology of SIVsm in US Primate Centers unravels the origin of SIVmac and SIVstm. J Virol 79: 8991–9005. 15994793 10.1128/JVI.79.14.8991-9005.2005 PMC1168739

[95] Chen Z, Telfier P, Gettie A, Reed P, Zhang L, et al (1996) Genetic characterization of new West African simian immunodeficiency virus SIVsm: geographic clustering of household-derived SIV strains with human immunodeficiency virus type 2 subtypes and genetically diverse viruses from a single feral sooty mangabey troop. J Virol 70: 3617–3627. 8648696 10.1128/jvi.70.6.3617-3627.1996 PMC190237

[96] Souquiere S, Bibollet-Ruche F, Robertson DL, Makuwa M, Apetrei C, et al (2001) Wild *Mandrillus sphinx* are carriers of two types of lentivirus. J Virol 75: 7086–7096. 11435589 10.1128/JVI.75.15.7086-7096.2001 PMC114437

[97] Pandrea I, Parrish NF, Raehtz K, Gaufin T, Barbian HJ, et al (2012) Mucosal SIV transmission in African green monkeys: Susceptibility to infection is proportional to target cell availability at mucosal sites. J Virol 86: 4158–4168. 22318138 10.1128/JVI.07141-11 PMC3318646

[98] Ma ZM, Keele BF, Qureshi H, Stone M, Desilva V, et al (2011) SIVmac251 is inefficiently transmitted to rhesus macaques by penile inoculation with a single SIVenv variant found in ramp-up phase plasma. AIDS Res Hum Retroviruses 27: 1259–1269. 21732792 10.1089/aid.2011.0090 PMC3227244

[99] Andelman SJ (1986) Ecological and social determinants of cercopithecine mating patterns. In: Rubenstein DI, Wrangham RW, editors. Ecological Aspects of Social Evolution. Princeton: Princeton University Press. pp. 201–216.

[100] Cords M (2000) The number of males in guenon groups. In: Kappeler P, editor. Primate Males: Causes and Consequences of Variation in Group Composition. Cambridge, UK: Cambridge University Press. pp. 84–96.

[101] Keddy AC (1986) Female mate choice in vervet monkeys (*Cercopithecus aethiops sabaeus*). American Journal of Primatology 10: 125–134. 10.1002/ajp.1350100204 31979492

[102] Weingrill T, Willems EP, Krutzen M, Noe R (2011) Determinants of Paternity Success in a Group of Captive Vervet Monkeys (*Chlorocebus aethiops sabaeus*). International Journal of Primatology 32: 415–429.

[103] Cowlishaw G, Dunbar RIM (1991) Dominance rank and mating success in male primates. Animal Behaviour 41: 1045–1056.

[104] Andelman SJ (1987) Evolution of concealed ovulation in vervet monkeys (*Cercopithecus aethiops*). Am Naturalist 129: 785–799.

[105] Nerrienet E, Amouretti X, Muller-Trutwin MC, Poaty-Mavoungou V, Bedjebaga I, et al (1998) Phylogenetic analysis of SIV and STLV type I in mandrills (*Mandrillus sphinx*): indications that intracolony transmissions are predominantly the result of male-to-male aggressive contacts. AIDS Res Hum Retroviruses 14: 785–796. 9643378 10.1089/aid.1998.14.785

[106] Cheney D, Seyfarth R (1983) Nonrandom dispersal in free-ranging vervet monkeys: Social and genetic consequences. Am Naturalist 122: 392–412.

[107] Amedee AM, Rychert J, Lacour N, Fresh L, Ratterree M (2004) Viral and immunological factors associated with breast milk transmission of SIV in rhesus macaques. Retrovirology 1: 17. 15253769 10.1186/1742-4690-1-17 PMC493286

[108] Chahroudi A, Meeker T, Lawson B, Ratcliffe S, Else J, et al (2011) Mother-to-infant transmission of simian immunodeficiency virus is rare in sooty mangabeys and is associated with low viremia. J Virol 85: 5757–5763. 21450815 10.1128/JVI.02690-10 PMC3126302

[109] Beer B, Denner J, Brown CR, Norley S, zur Megede J, et al (1998) Simian immunodeficiency virus of African green monkeys is apathogenic in the newborn natural host. J Acquir Immune Defic Syndr Hum Retrovirol 18: 210–220. 9665497 10.1097/00042560-199807010-00003

[110] Ruprecht RM, Baba TW, Liska V, Ray NB, Martin LN, et al (1999) Oral transmission of primate lentiviruses. J Infect Dis 179 Suppl 3: S408–412. 10099108 10.1086/314794

[111] Chenine AL, Siddappa NB, Kramer VG, Sciaranghella G, Rasmussen RA, et al (2010) Relative transmissibility of an R5 clade C simian-human immunodeficiency virus across different mucosae in macaques parallels the relative risks of sexual HIV-1 transmission in humans via different routes. J Infect Dis 201: 1155–1163. 20214475 10.1086/651274 PMC2838976

[112] Cheney D, Seyfarth R, Smuts B (1986) Social relationships and social cognition in nonhuman primates. Science 234: 1361–1366. 3538419 10.1126/science.3538419

[113] Schwab P, Debes PV, Witt T, Hartl GB, Hmwe SS, et al (2012) Genetic structure of the common impala (*Aepyceros melampus melampus*) in South Africa: pylogeography and implications for conservation. J Zool Syst Evol Res 50: 76–84.

[114] Sithaldeen R, Bishop JM, Ackermann RR (2009) Mitochondrial DNA analysis reveals Plio-Pleistocene diversification within the chacma baboon. Mol Phylogenet Evol 53: 1042–1048. 19665055 10.1016/j.ympev.2009.07.038

[115] Drummond AJ, Suchard MA, Xie D, Rambaut A (2012) Bayesian Phylogenetics with BEAUti and the BEAST 1.7. Mol Biol Evol 29: 1969–1973. 22367748 10.1093/molbev/mss075 PMC3408070

[116] Pandrea I, Onanga R, Rouquet P, Bourry O, Ngari P, et al (2001) Chronic SIV infection ultimately causes immunodeficiency in African non-human primates. AIDS 15: 2461–2462. 11826852 10.1097/00002030-200112070-00019

[117] Traina-Dorge V, Blanchard J, Martin L, Murphey-Corb M (1992) Immunodeficiency and lymphoproliferative disease in an African green monkey dually infected with SIV and STLV-I. AIDS Res Hum Retroviruses 8: 97–100. 1310605 10.1089/aid.1992.8.97

[118] Ling B, Apetrei C, Pandrea I, Veazey RS, Lackner AA, et al (2004) Classic AIDS in a sooty mangabey after an 18-year natural infection. J Virol 78: 8902–8908. 15280498 10.1128/JVI.78.16.8902-8908.2004 PMC479084

[119] Apetrei C, Gormus B, Pandrea I, Metzger M, ten Haaft P, et al (2004) Direct inoculation of simian immunodeficiency virus from sooty mangabeys in black mangabeys (*Lophocebus aterrimus*): first evidence of AIDS in a heterologous African species and different pathologic outcomes of experimental infection. J Virol 78: 11506–11518. 15479792 10.1128/JVI.78.21.11506-11518.2004 PMC523258

[120] Etienne L, Nerrienet E, LeBreton M, Bibila GT, Foupouapouognigni Y, et al (2011) Characterization of a new simian immunodeficiency virus strain in a naturally infected *Pan troglodytes troglodytes* chimpanzee with AIDS related symptoms. Retrovirology 8: 4. 21232091 10.1186/1742-4690-8-4 PMC3034674

[121] Pandrea I, Cornell E, Wilson C, Ribeiro RM, Ma D, et al (2012) Coagulation biomarkers predict disease progression in SIV-infected nonhuman primates. Blood 120: 1357–1366. 22653975 10.1182/blood-2012-03-414706 PMC3423778

[122] Lederer S, Favre D, Walters KA, Proll S, Kanwar B, et al (2009) Transcriptional profiling in pathogenic and non-pathogenic SIV infections reveals significant distinctions in kinetics and tissue compartmentalization. PLoS Pathog 5: e1000296. 19214219 10.1371/journal.ppat.1000296 PMC2633618

[123] Lederman MM, Alter G, Daskalakis DC, Rodriguez B, Sieg SF, et al (2010) Determinants of protection among HIV-exposed seronegative persons: an overview. J Infect Dis 202 Suppl 3: S333–338. 20887220 10.1086/655967 PMC3184646

[124] Watkins DI, Burton DR, Kallas EG, Moore JP, Koff WC (2008) Nonhuman primate models and the failure of the Merck HIV-1 vaccine in humans. Nat Med 14: 617–621. 18535579 10.1038/nm.f.1759 PMC3697853

[125] Grobler P, Turner T (2010) A novel trap design for the capture and sedation of vervet monkeys (*Chlorocebus aethiops*). South Afr J Wildlife Res 40: 163–168.

[126] Bibollet-Ruche F, Bailes E, Gao F, Pourrut X, Barlow KL, et al (2004) A new simian immunodeficiency virus lineage (SIVdeb) infecting de Brazza's monkeys (*Cercopithecus neglectus*): Evidence for a Cercopithecus monkey virus clade. J Virol 78: 7748–7762. 15220449 10.1128/JVI.78.14.7748-7762.2004 PMC434087

[127] Gao F, Yue L, Robertson DL, Hill SC, Hui H, et al (1994) Genetic diversity of human immunodeficiency virus type 2: evidence for distinct sequence subtypes with differences in virus biology. J Virol 68: 7433–7447. 7933127 10.1128/jvi.68.11.7433-7447.1994 PMC237186

[128] Jin MJ, Rogers J, Phillips-Conroy JE, Allan JS, Desrosiers RC, et al (1994) Infection of a yellow baboon with simian immunodeficiency virus from African green monkeys: evidence for cross-species transmission in the wild. J Virol 68: 8454–8460. 7966642 10.1128/jvi.68.12.8454-8460.1994 PMC237322

[129] Edgar RC (2004) MUSCLE: multiple sequence alignment with high accuracy and high throughput. Nucleic Acids Res 32: 1792–1797. 15034147 10.1093/nar/gkh340 PMC390337

[130] Guindon S, Dufayard JF, Lefort V, Anisimova M, Hordijk W, et al (2010) New algorithms and methods to estimate maximum-likelihood phylogenies: assessing the performance of PhyML 3.0. Syst Biol 59: 307–321. 20525638 10.1093/sysbio/syq010

[131] Huson DH, Bryant D (2006) Application of phylogenetic networks in evolutionary studies. Mol Biol Evol 23: 254–267. 16221896 10.1093/molbev/msj030

[132] Simon F, Souquiere S, Damond F, Kfutwah A, Makuwa M, et al (2001) Synthetic peptide strategy for the detection of and discrimination among highly divergent primate lentiviruses. AIDS Res Hum Retroviruses 17: 937–952. 11461679 10.1089/088922201750290050

[133] Kuiken C, Foley B, Leitner T, Apetrei C, Hahn B, et al.. (2010) HIV Sequence Compendium. Los Alamos National Laboratory, New Mexico, LA-UR 10-03684: Theoretical Biology and Biophysics Group.

