# Supplementary figures and images for: SIVagm Infection in Wild African Green Monkeys from South Africa: Epidemiology, Natural History, and Evolutionary Considerations
Source: PLoS Pathog. 2013 Jan 17;9(1):e1003011. doi: 10.1371/journal.ppat.1003011 (PMC3547836; doi:10.1371/journal.ppat.1003011)

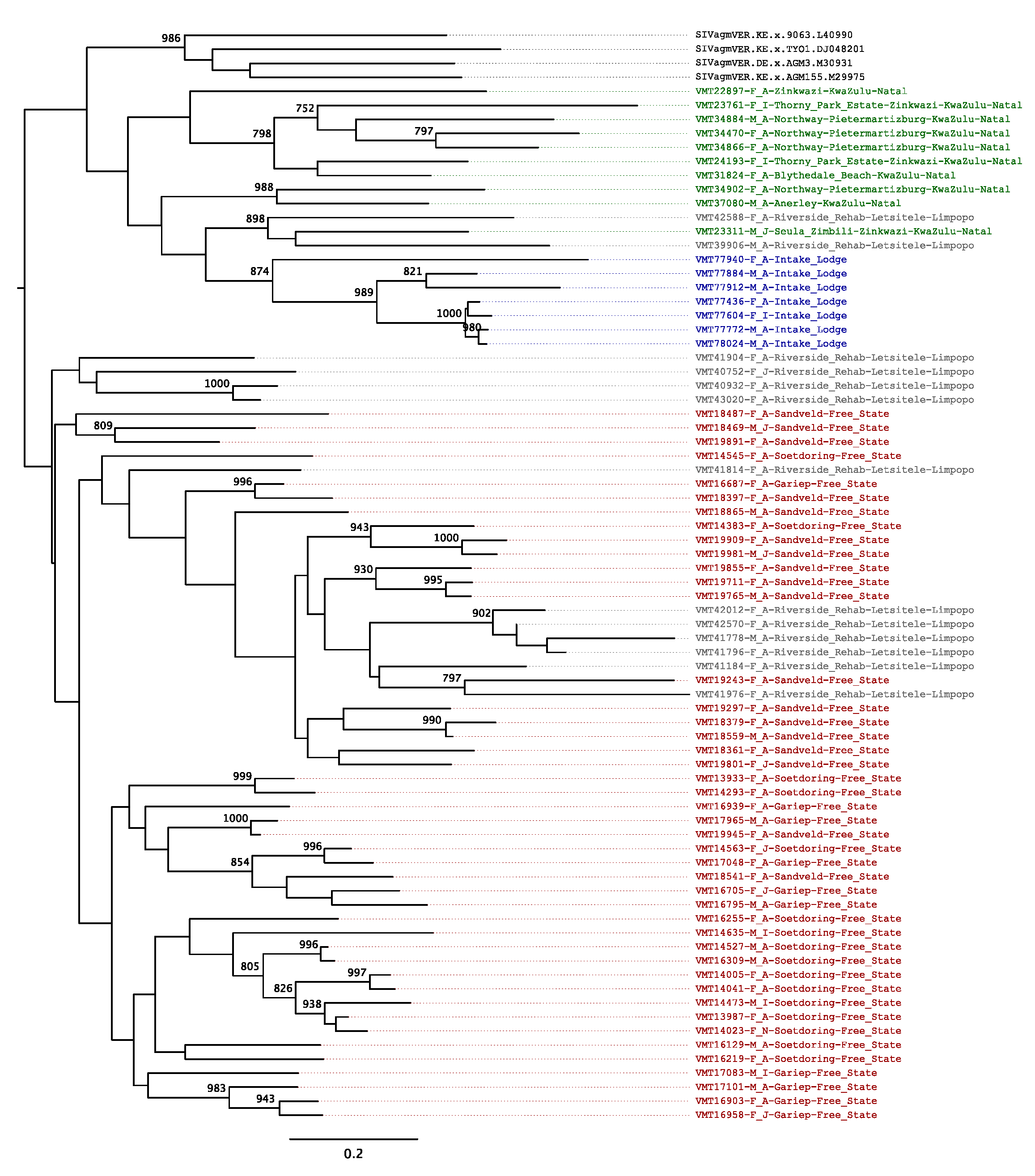

Supplement: Figure S1 — Maximum likelihood trees for env gene of the newly derived SIVagmVer sequences from wild vervets in South Africa. Maximum likelihood estimates were performed using 1000 replicate bootstrap analysis. Internal nodes indicate level of support values for internal branching. Sequences are colored depending on the region in which the sequences were sampled, with red indicating sequences from Free State, green indicating sequences from KwaZulu-Natal, and blue indicating sequences from Eastern Coast territories. In addition, sequences colored in gray are sampled from the Riverside Wildlife Rehabilitation and Environmental Education Centre (RWREC), Letsitele, Limpopo semifree colony that is housing released pets or animals with health problems, and their origin is unknown, and sequences colored in black indicate outgroup sequences. The strain nomenclature includes the identification number, monkey status (M-male, F-female; I-infant; J-juvenile and A-adult), and the site and the state of origin (FS-Free State; KZN-KwaZulu Natal; EC-Eastern Cape). (TIFF) [file ppat.1003011.s001.tiff]

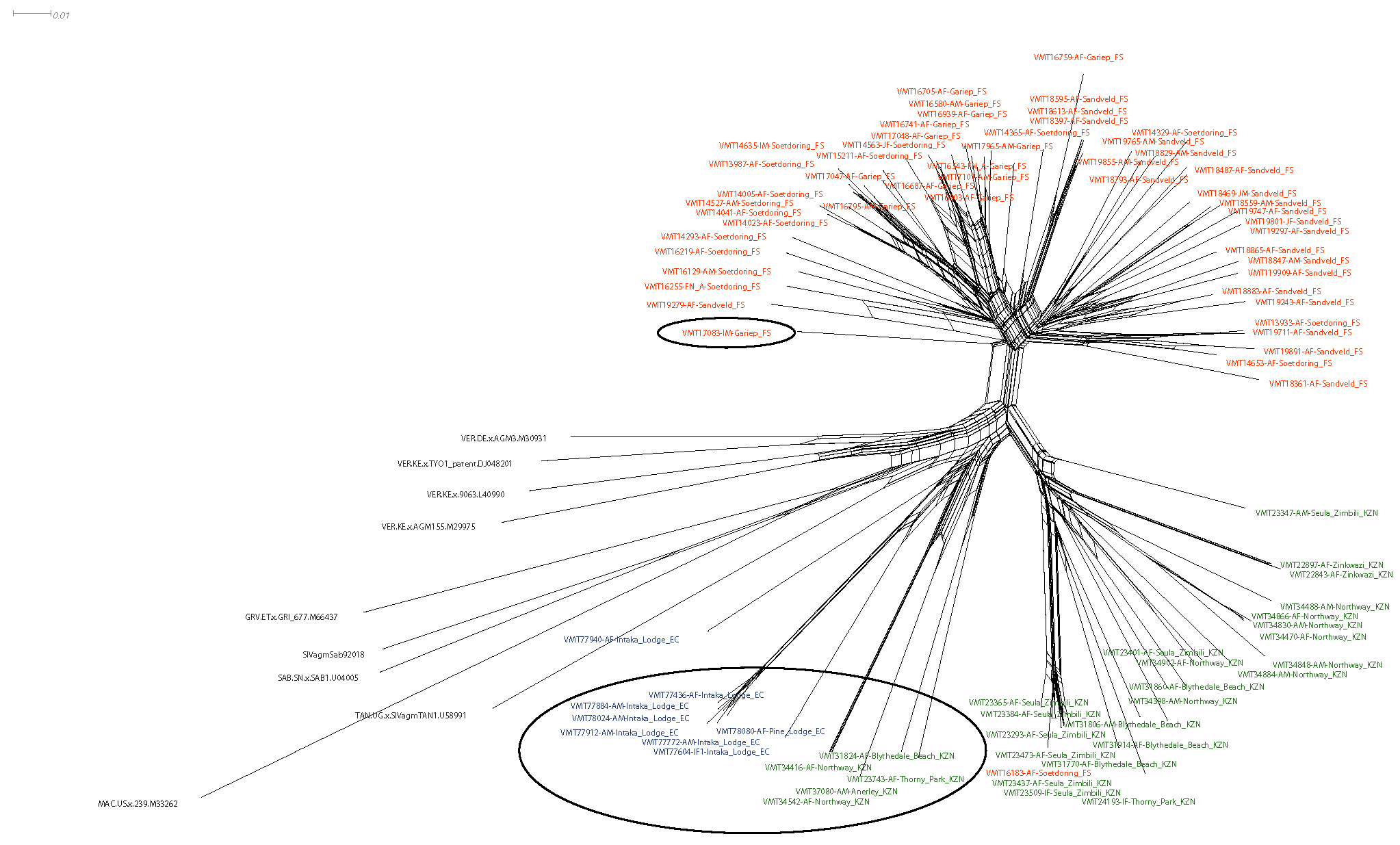

Supplement: Figure S2 — SplitsTree showing the pol gene of South African SIVagmVer sequences. Nodes are colored by the region in which they were sampled, with red, blue and green denoting Free State, East Coast and KwaZulu-Natal, respectively. Potentially interesting recombinants have been circled, including VMT17083-IM-Gariep_FS and a cluster indicating possible mixing between East Coast and KwaZulu-Natal sequences. (TIFF) [file ppat.1003011.s002.tiff]

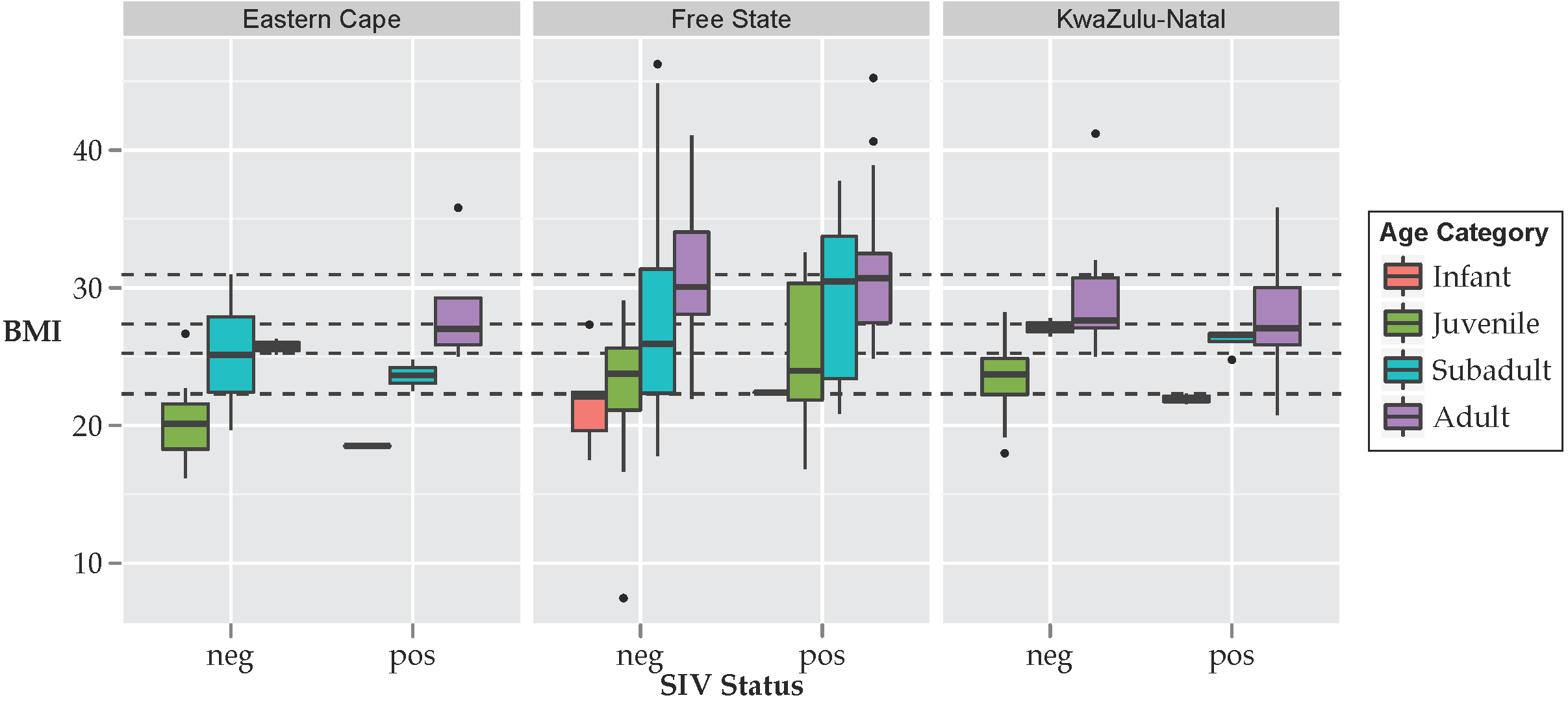

Supplement: Figure S3 — Comparative body mass index (BMI) assessment based on age and location in SIV-infected and SIV uninfected wild vervet monkeys ( Chlorocebus pygerithrus ) from South Africa. (TIFF) [file ppat.1003011.s003.tiff]
