# Supplementary material for: SIVagm Infection in Wild African Green Monkeys from South Africa: Epidemiology, Natural History, and Evolutionary Considerations
Source: PLoS Pathog. 2013 Jan 17;9(1):e1003011. doi: 10.1371/journal.ppat.1003011 (PMC3547836; doi:10.1371/journal.ppat.1003011)
Supplement: Table S1 — SIV prevalence in vervet monkeys from South Africa based on location of collected samples. (DOC) [file ppat.1003011.s004.doc]

| Province | Location | Total tested | Total SIV-infected | SIVprevalence |
| --- | --- | --- | --- | --- |
| **Eastern Cape** |  | 23 | 8 | 34.8 |
|  | **Intake Lodge** | 13 | 7 | 53.8 |
|  | **NMMU Reserve** | 6 | 0 | 0 |
|  | **Pine Lodge** | 4 | 1 | 25 |
| **Free State** |  | 147 | 65 | 44.9 |
|  | **Sandveld** | 47 | 28 | 59.6 |
|  | **Soetdoring** | 53 | 21 | 39.6 |
|  | **Gariep** | 47 | 16 | 33.3 |
| **KwaZulu Natal** |  | 53 | 30 | 59.2 |
|  | **Anerley** | 3 | 1 | 33.3 |
|  | **Blytedale Beach** | 13 | 5 | 38.5 |
|  | **Kwela** | 13 | 11 | 84.6 |
|  | **Seula Zimbili** | 10 | 8 | 80 |
|  | **Thorny Park** | 4 | 2 | 50 |
|  | **Zinkwazi** | 10 | 3 | 33.3 |
| **Total** |  | 223 | 103 | 45.8 |
