# Supplementary material for: SIVagm Infection in Wild African Green Monkeys from South Africa: Epidemiology, Natural History, and Evolutionary Considerations
Source: PLoS Pathog. 2013 Jan 17;9(1):e1003011. doi: 10.1371/journal.ppat.1003011 (PMC3547836; doi:10.1371/journal.ppat.1003011)
Supplement: Table S2 — Age- and sex-related prevalence of SIVagmVer in semifree vervets from Riverside Wildlife Rehabilitation and Education Center, Letsitele, Limpopo. (DOC) [file ppat.1003011.s005.doc]

|  |  | **Females** |  |  | **Males** |  |
| --- | --- | --- | --- | --- | --- | --- |
|  | **Total** | **SIV+** | **Prevalence (%)** | **Total** | **SIV+** | **Prevalence (%)** |
| **Infants** | **10** | **0** | **0** | **16** | **0** | **0** |
| **Juveniles** | **11** | **2** | **18** | **20** | **0** | **0** |
| **Adults** | **42** | **10** | **24** | **39** | **5** | **13** |
