# Supplementary material for: SIVagm Infection in Wild African Green Monkeys from South Africa: Epidemiology, Natural History, and Evolutionary Considerations
Source: PLoS Pathog. 2013 Jan 17;9(1):e1003011. doi: 10.1371/journal.ppat.1003011 (PMC3547836; doi:10.1371/journal.ppat.1003011)
Supplement: Table S3 — Estimates of evolutionary divergence over sequence pairs between groups. The number of base substitutions per site±standard error estimate(s) from averaging over all sequence pairs between groups are shown± Analyses were conducted using the Maximum Composite Likelihood model [1]. The rate variation among sites was modeled with a gamma distribution (shape parameter = 0.5). env analysis involved 81 nucleotide sequences. All positions containing gaps and missing data were eliminated. There were a total of 822 positions in the final dataset for env. pol analysis involved 98 nucleotide sequences. All positions containing gaps and missing data were eliminated. There were a total of 574 positions in the final pol dataset. Evolutionary analyses were conducted in MEGA5 [2]. (DOC) [file ppat.1003011.s006.doc]

*env*

|  | **Outgroup** | **Free State** | **KwaZulu Natal** | **Eastern Cape** |
| --- | --- | --- | --- | --- |
| **Outgroup** |  |  |  |  |
| **Free State** | 50.3±4.2 |  |  |  |
| **KwaZulu Natal** | 51.3±4.3 | 36.6±3.1 |  |  |
| **Eastern Cape** | 51.1±4.3 | 24.3±1.8 | 33.2±2.9 |  |

pol

|  | **Outgroup** | **Free State** | **KwaZulu Natal** | **Eastern Cape** |
| --- | --- | --- | --- | --- |
| **Outgroup** |  |  |  |  |
| **Free State** | 53.6±5 |  |  |  |
| **KwaZulu Natal** | 54.7±5.1 | 27.1±2.5 |  |  |
| **Eastern Cape** | 53.5±5.5 | 33±3.6 | 33.3±3.6 |  |
