# Supplementary material for: SIVagm Infection in Wild African Green Monkeys from South Africa: Epidemiology, Natural History, and Evolutionary Considerations
Source: PLoS Pathog. 2013 Jan 17;9(1):e1003011. doi: 10.1371/journal.ppat.1003011 (PMC3547836; doi:10.1371/journal.ppat.1003011)
Supplement: Table S4 — Most recent common ancestor (MRCA) estimates using relaxed molecular clocks to incorporate biogeographical assumptions. Calibration for the env gene is based on time to MRCA (TMRCA) of SIVagm sequences sampled around the Drakensberg Mountains, presumed to be 100,000–3,000,000 years of age. Calibration of the pol gene is based on TMRCA of SIVagm sequences as before, as well as SIVdrl sequences sampled from the Bioko Island of Equatorial Guinea, presumed to have been isolated for approximately 10,000 years. These estimates indicate that SIV is potentially considerably older than previous estimates, and refute timing estimates based exclusively on molecular clocks without calibration. (DOC) [file ppat.1003011.s007.doc]

| **TMRCA** | ***env* (SIVagm) mean Yrs (95% HPD)** | ***pol* (SIVagm+SIVdrl) mean Yrs (95% HPD)** |
| --- | --- | --- |
| SIV | 789,650 (150,890–2,594,700) | 247,680 (1894,90–316,750) |
| SIVagm South Africa | 329,350 (10,000–1,076,600) | 104,120 (10,000–112,480) |
| SIVagm East Cape | 180,120 (37,882–593,520) | 68467 (43,623–79,077) |
| SIVagm KwaZulu Natal | 294,920 (75,998–960,300) | 103,820 (10,000–114,630) |
| SIVagm Free State | 231,780 (59,343-761,630) | 84,193 (62,547-106,450) |
| SIVblc^1^ Bioko | - | 37,126 (18,600-58,423) |
| SIVdrl^2^ Bioko | - | 6,239 (3,811-8,774) |
| SIVprg^3^ Bioko | - | 44,567 (18,934-72,560) |
| SIVreg^4^ Bioko | - | 60,789 (42,896-79,077) |

^1^SIVblc-SIV naturally infecting the black colobus (*Colobus satanas*); ^2^SIVdrl-SIV naturally infecting the drill monkey (*Mandrillus leucophaeus*); ^3^SIVprg-SIV naturally infecting the Preuss’s monkey (*Cercopithecus preusi*); ^4^SIVreg-SIV naturally infecting the red-eared monkey (*C. erithrotis*).
