# Supplementary material for: SIVagm Infection in Wild African Green Monkeys from South Africa: Epidemiology, Natural History, and Evolutionary Considerations
Source: PLoS Pathog. 2013 Jan 17;9(1):e1003011. doi: 10.1371/journal.ppat.1003011 (PMC3547836; doi:10.1371/journal.ppat.1003011)
Supplement: Text S1 — Details on animal origin, animal capture, sampling methodology and clinical assessment. (DOC) [file ppat.1003011.s008.doc]

**SIVagm Infection in Wild African Green Monkeys from South Africa: Epidemiology, Natural History and Evolutionary Considerations**

Dongzhu Ma^1‡^, Anna Jasinska^2‡^, Jan Kristoff^1^, J. Paul Grobler^3^, Trudy Turner^3,4^, Yoon Jung^2^, Christopher Schmitt ^2^, Kevin Raehtz^1^, Felix Feyertag^5^, Natalie Martinez Sosa^1^, Viskam Wijewardana^1^, Donald S. Burke^1^, David L. Robertson^5^, Russell Tracy^6^, Ivona Pandrea^1,7^, Nelson Freimer^2^, and Cristian Apetrei^1,8*^ for "The International Vervet Research Consortium"

**Text S1**

**Materials and Methods**

**Sample collection**

Animal origin. All vervets captured and sampled in this study were free ranging, from habitats that varied in both landscape and levels of anthropogenic impact. The majority of locations were nature reserves or areas around game lodges in which vervet groups had very little contact with humans and little to no provisioning (e.g., Sandveld, Soetdoring, NMMU, Pine, Intake). The rest of the sites were at lodges or on farms in human-inhabited areas (e.g., Gariep, all sites in KwaZulu-Natal). Sandveld and Soetdoring are in what is known as veld habitat, or relatively flat, open grass and scrublands. Eastern Cape, all other Free State locations, and Thorny Park Estate are located near riparian scrublands and forests adjoining farmlands and human-maintained pastures. Camp Anerley, Zinkwazi Lagoon Lodge, Blythedale Beach, Kwela Lodge (Pietermaritzburg), and Seula Zimbili are all in highly anthropogenic landscapes with some tree cover.

Although the proportion of animals sampled in each group cannot be reliably estimated due to the short-term nature of sampling time within each population, our past studies using the same trapping method report capture of 50-90% of monkeys in the groups targeted . Our goal was to sample as many animals from each group as possible, and trapping continued at each site until animals stopped going into traps (typically after the second day of trapping).

To confirm that the sample collection is representative of the wild AGM population, we included in our study a group of 138 samples collected from AGMs originating from the Riverside Wildlife Rehabilitation and Environmental Education Centre (RWREC) located in the Limpopo area. While vervets are semifree at this location, RWREC is a temporary sanctuary for AGMs that have been taken out of the wild and kept in captivity or used as pets; thus, SIV prevalence and incidence rates in AGMs from the RWREC are likely to be different from those observed in wild animals.

Animal capture. Groups of vervet monkeys were located by conferral with local informants, and provisioned for 3-5 days with a novel food item (e.g., oranges, maize). Drop traps were moved into an area where animals were seen to reliably feed on bait, preferably near observed sleeping sites. Drop traps consisted of a one meter square wooden frame with a wire mesh top and internal squeeze cage mechanism, which was propped up by a trigger mechanism that caused the trap to fall once bait directly under the trap was moved (see for trap specifications and schematics). Traps were ideally placed under trees that provide shade to avoid heat distress during trapping. Once animals were observed feeding regularly under propped up (but not set) traps, traps were set early the following morning, so that animals would enter them after waking. Traps were allowed to sit for one hour without disruption so as not to scare the group away, after which time two researchers approached each trap individually and used the internal partition to move the animal to one side of the trap. It should be noted that animals do not become agitated in the traps unless a person approaches. Animals not subject to immediate removal from traps were monitored closely for signs of distress. While restrained in the trap, monkeys were anesthetized with **Zoletil or Ketamine (10mg/kg)** injected intramuscularly into the thigh. Members of our team have used this method of collection for over 30 years to sample populations in Ethiopia, Kenya, South Africa, Botswana and Ghana .

Sampling. Plasma samples used in this study come from the Systems Biology Sample Repository, which consists of biological samples and phenotypic data collected from >1500 AGMs from different geographic locations . Briefly, multiple blood samples for various analyses were drawn from sedated animals through femoral venipuncture using a SafetyLock System (Becton Dickinson, Franklin Lakes, NJ) within 5-10 min after administration of **Zoletil. B**lood was drawn into the vacutainer tubes containing EDTA as anticoagulant (BD) and mixed by inverting the tube several times. To collect the plasma fraction, whole blood was centrifuged at 1,300-1,500xg for 10 mins in a portable centrifuge (E8F Lab Essentials, Inc, **Monroe, GA**). Under the field conditions only a simple sample spin without the deceleration control needed for PBMC separation was possible.

Clinical assessment. A veterinarian experienced with nonhuman primates performed physical exams for all the AGMs trapped and sampled in this study. Clinical signs were noted during this physical exam: general condition, muscle mass, condition of pelage, dehydration, ectoparasites, dental and gum decay, lesions.

**Results**

Sample representativity. We appreciate that the samples collected are representative of the wild AGM populations: AGMs were not targeted for trapping; traps were left open and the animals entered them freely. Usually, juveniles and females enter traps before adult males. We acknowledge that the most likely source of sample bias that could be caused by animal health include: (i) sick animals need the food more, and so will be less wary and more easily captured, or (ii) healthy animals out-compete the sick animals and so are more easily captured. Given that large adult males are often trapped last in each group, after smaller bodied adult females and young juveniles, the latter case is unlikely. In the former case, it is important to note that in most wildlife studies of disease, health and body condition are assumed not to influence trapping or capture bias to the point of skewing results . One study explicitly tested this, wherein researchers found that in bears trapped using a trap/bait method (as opposed to being darted) during a long term mark-recapture study, those with poorer health – as measured by overall body condition – did not trap any more or less frequently than animals with better health . Therefore, we believe that it is unlikely that health or body condition adversely affected trap rates.

To validate our capture strategy, we assessed the prevalence of SIVagm infection in a semi-free colony of AGMs. At the RWREC, monkeys previously kept as pets are released in a semifree environment and, while a social system is established, the ecosystem is different from that of wild animals, where the alliances and affiliations are strongly influenced by kinship, and female juveniles frequently acquire the rank and social status of their mothers. Testing of AGMs at the RWREC showed significantly lower levels of SIVagm prevalence (Table S1) compared to that observed at all other locations in the wild (Table S2): 19% (12/63) in females and 7% (5/75) in males. Similar to wild animals, the virus was unevenly distributed among age groups: 0% (0/26) in infants, 7% (2/31) in juveniles (males: 0%; females: 18%, 2/11) and 19% (15/81) in adults (males: 13%, 5/39; females: 24%, 10/42) (Table S1). Furthermore, in keeping with the origin of monkeys, which are collected as pets from every region of South Africa and mixed at the RWREC, SIVagm strains identified in AGMs from the RWREC do not cluster together in the phylogenetic trees, but are interspersed among all the clusters of strains from wild animals (Figure S1).

The differences in SIV prevalence levels between AGMs at the RWREC and in the wild (14% *vs*. 45%) thus validate our capture strategy in the wild. Furthermore, results obtained at the RWREC demonstrate that the study of SIV spread in natural hosts from colonies and sanctuaries cannot be a proper substitute for the study of SIV spread in the wild.

Clinical data. Physical exam did not reveal any clinical sign of immunodeficiency (failure to thrive or opportunistic infections), with the exception of an adult female in one of the Free State parks that had lesions on her vulva which might have been herpes sores. Although we did not perform a follow-up study on trapped individuals, we compared BMI, body weight and waist circumference between SIV-positive and SIV-negative individuals, controlling for age, sex and location, which did not show significant differences between the groups. Distribution of BMIs between SIV-positive and SIV-negative monkeys indicates that SIV status does not have an impact on normal weight. We attempted to estimate weight loss cross-sectionally by comparing the body mass indices of SIV-positive individuals to SIV-negative individuals while controlling for age, sex, and trap location using a linear mixed-effects model on BMI, body weight, and waist circumference. Although overall BMI within age classes was lower in SIV-positive than in SIV-negative individuals, that relationship was lost once we controlled for age, sex, and location (Figure S2). Similarly, body weight and waist circumference showed no significant differences between SIV-positive and SIV-negative individuals once age, sex, and provenance were controlled.

**References**

1. Grobler P, Turner T (2010) A novel trap design for the capture and sedation of vervet monkeys (*Chlorocebus aethiops*). South Afr J Wildlife Res 40: 163-168.

2. Brett FL, Turner TR, Jolly CJ, Cauble RG (1982) Trapping baboons and vervet monkeys from wild, free-ranging populations. J Wildl Manage 46: 164-174.

3. Jasinska AJ, Lin MK, Service S, Choi OW, Deyoung J, et al. (2012) A non-human primate system for large-scale genetic studies of complex traits. Hum Mol Genet 21: 3307-3316.

4. Cattet M, Boulanger J, Stenhouse G, Powell RA, Reynolds-Hogland MJ (2008) An evaluation of long-term capture effects in ursids: Implications for wildlife welfare and research. J Mammalogy 89: 973-990.
